# Peripheral gating of mechanosensation by glial diazepam binding inhibitor

**DOI:** 10.1172/JCI176227

**Published:** 2024-06-18

**Authors:** Xinmeng Li, Arthur Silveira Prudente, Vincenzo Prato, Xianchuan Guo, Han Hao, Frederick Jones, Sofia Figoli, Pierce Mullen, Yujin Wang, Raquel Tonello, Sang Hoon Lee, Shihab Shah, Benito Maffei, Temugin Berta, Xiaona Du, Nikita Gamper

**Affiliations:** 1 Department of Pharmacology; The Key Laboratory of Neural and Vascular Biology, Ministry of Education; The Key Laboratory of New Drug Pharmacology and Toxicology, Hebei Medical University, Shijiazhuang, Hebei, China.; 2 Pain Research Center, Department of Anesthesiology, University of Cincinnati Medical Center, Cincinnati, Ohio, USA.; 3 Faculty of Biological Sciences, School of Biomedical Sciences, University of Leeds, Leeds, United Kingdom.; 4 Department of Life Sciences, Manchester Metropolitan University, Manchester, United Kingdom.

**Keywords:** Neuroscience, Ion channels, Pain, Peptides

## Abstract

We report that diazepam binding inhibitor (DBI) is a glial messenger mediating crosstalk between satellite glial cells (SGCs) and sensory neurons in the dorsal root ganglion (DRG). DBI is highly expressed in SGCs of mice, rats, and humans, but not in sensory neurons or most other DRG-resident cells. Knockdown of DBI results in a robust mechanical hypersensitivity without major effects on other sensory modalities. In vivo overexpression of DBI in SGCs reduces sensitivity to mechanical stimulation and alleviates mechanical allodynia in neuropathic and inflammatory pain models. We further show that DBI acts as an unconventional agonist and positive allosteric modulator at the neuronal GABA_A_ receptors, particularly strongly affecting those with a high-affinity benzodiazepine binding site. Such receptors are selectively expressed by a subpopulation of mechanosensitive DRG neurons, and these are also more enwrapped with DBI-expressing glia, as compared with other DRG neurons, suggesting a mechanism for a specific effect of DBI on mechanosensation. These findings identified a communication mechanism between peripheral neurons and SGCs. This communication modulates pain signaling and can be targeted therapeutically.

## Abstract 2

Satellite glial cells release an endogenous benzodiazepine-like peptide that acts on the intrinsic GABAergic inhibitory system of the dorsal root ganglion to gate mechanical pain.

## Graphical abstract

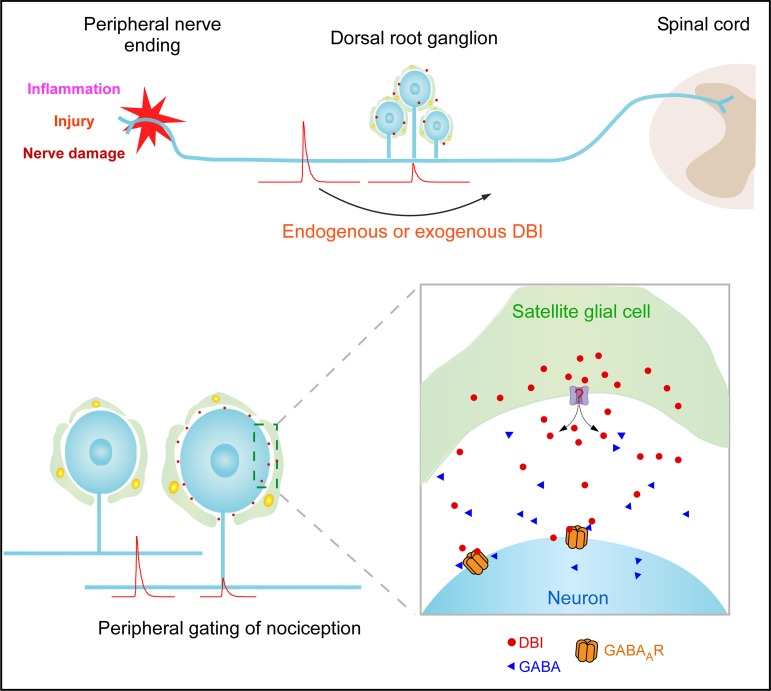

## Introduction

Despite remarkable progress in our understanding of the fundamental biology of pain, current therapies for chronic pain are often inadequate and prone to serious side effects within the central nervous system (CNS). Hence, there is an increasing focus on the peripheral nociceptive pathways (1). Specifically, peripheral somatosensory ganglia, such as the dorsal root ganglion (DRG), emerge as an early gate within the somatosensory system (2–5). Because of the pseudounipolar morphology of the DRG neurons, the action potentials traveling from the peripheral nerve terminals to the spinal cord need to pass through the axonal bifurcations (T-junctions) at the DRG, where a propagating spike could fail (2, 3, 6–10). Accumulating evidence suggests that such failure does occur physiologically; moreover, it can be dynamically regulated, manifesting as filtering of the throughput firing frequency at the DRG (2–4, 6, 11). Although fundamentals of such filtering are only beginning to emerge, one revealed mechanism utilizes the ganglion’s intrinsic inhibitory GABAergic system (2, 3, 12). Indeed, peripheral sensory neurons abundantly express GABA_A_ receptors (reviewed in ref. 13), and some of them are capable of producing and releasing GABA (2, 3, 14). Accordingly, direct ganglionic injections of GABA_A_ agonists (2, 15) or GABA reuptake inhibitor (2, 16) provide strong relief of acute and chronic pain. Direct electrophysiological measurements of spike propagation through the DRG revealed that GABA applied to DRG induces spike filtering in the nociceptive fibers, while administration of GABA_A_ antagonists reduces such filtering (3). These reports point to the existence of an inhibitory GABAergic tone at the DRG; this tone contributes to the ganglionic spike filtering, and it can be scaled up or down. Yet it is still unclear how transneuronal communication within the DRG is organized, especially given the fact that DRG neuron somata are individually wrapped by satellite glial cells (SGCs) (17). Being a physical barrier between the sensory neuron somata, SGCs are increasingly recognized as important actors in ganglionic communication (18–23). SGCs express receptors for some of the molecules released by the sensory neurons, particularly the purinergic P2Y receptors activated by ATP (20, 24). In turn, SGCs can also release neuroactive molecules, including ATP, chemokines, and cytokines (IL-1β, TNF-α) (23), which enable them to communicate with neurons and other SGCs. Additionally, SGCs are interconnected through gap junctions, especially connexin-43 (23), enabling complex SGC-to-SGC and SGC-to-neuron communications.

Here we report that SGCs abundantly produce and release a peptide called the diazepam binding inhibitor (DBI), also known as acyl-CoA–binding protein (ACBP). DBI is an 86–amino acid peptide belonging to the group of endogenous compounds exerting benzodiazepine-like effects, endozepines (25). DBI was discovered in the search for endogenous GABA_A_ receptor modulators; it competed with [^3^H]diazepam for binding to crude synaptic membranes from rat cerebral cortex (26–29). DBI is primarily expressed in astrocytes in the brain, especially in the olfactory bulb, hypothalamus, and hippocampus (30); moreover, *Dbi* is one of the most transcribed genes in astrocytes (31). DRG-resident SGCs share many features of the CNS’s astrocytes (23, 32); expression of DBI in SGCs was also reported (33, 34). Accumulating evidence suggests that DBI acts as an endogenous GABA_A_ receptor modulator, binding to the benzodiazepine binding site of the GABA_A_ receptors to allosterically modulate its activity (25). Neuronal action of DBI is still under investigation, but both positive (PAM) and negative (NAM) allosteric modulation on GABA_A_ receptors was reported (25, 35, 36). Given the emerging strong role of the GABA system in the control of peripheral nociceptive signaling, we investigated a potential role of DBI in nociception. We report that DBI, acting within DRGs, strongly antagonizes nociception induced by mechanical stimulation of the skin in vivo. Moreover, genetic overexpression of DBI antagonizes mechanical allodynia in the models of chronic inflammatory and neuropathic pain. Our data suggest that DBI acts as an unconventional agonist and a PAM at endogenous GABA_A_ receptors in the mechanosensitive neurons within the ganglion. Such action enhances peripheral ganglionic “gating” of the mechanosensory input to the CNS. Our findings revealed a component in the somatosensory system’s peripheral gate machinery and may point toward new types of analgesia.

## Results

### DBI is abundantly expressed in mouse, rat, and human SGCs.

**Figure 1:**
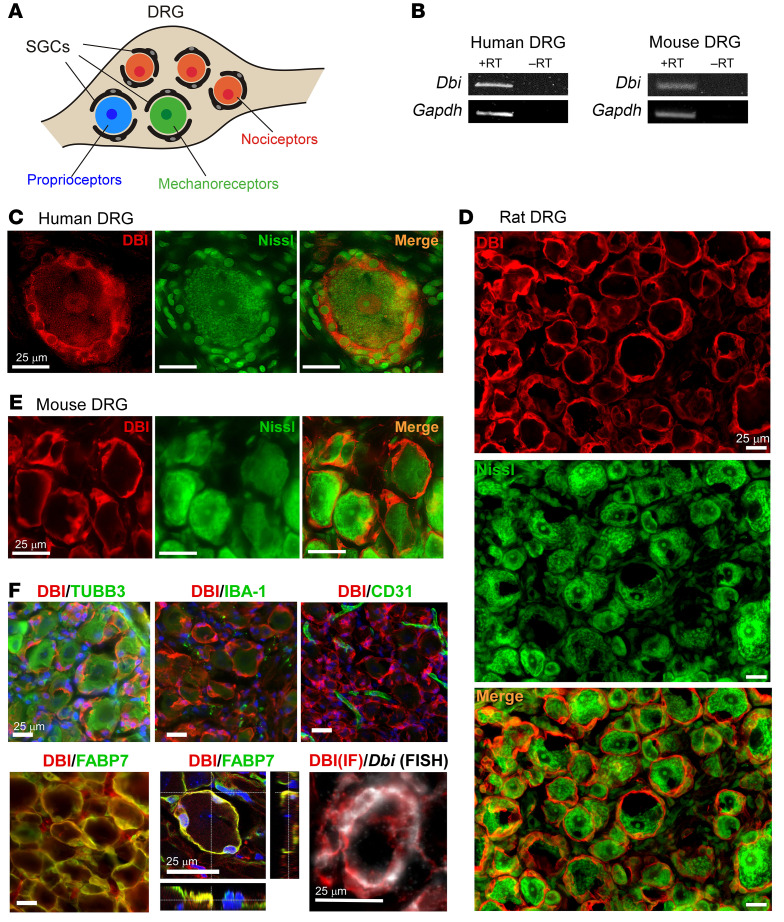
DBI is a satellite glial cell marker. (**A**) Schematic of superposition of sensory neuron somata and satellite glial cells (SGCs) within the dorsal root ganglion (DRG). (**B**) Detection of *Dbi* mRNA expression in human and mouse DRG by reverse transcription (RT) PCR. (**C**–**E**) Sections of human (**C**), rat (**D**), and mouse (**E**) DRGs with sensory neuron somata identified with Nissl (green) staining; DBI immunofluorescence (red) forms a characteristic pattern consistent with SGC wrapping. (**F**) Colabeling of mouse DRG sections with DBI (red) and markers of DRG-resident cell types (all in green): TUBB3 (pan-neuronal marker), IBA-1 (macrophages), CD31 (endothelial cells), and FABP7 (SGCs). Bottom middle panel shows confocal orthogonal view of DBI (red) and FABP7 (green) colabeling of SGCs wrapping around a single sensory neuron somata; blue is DAPI. Bottom right panel shows colabeling of DBI immunofluorescence with the *Dbi* transcript (white) using FISH. All scale bars: 25 μm.

First, we tested the expression of DBI within the DRG (schematized in Figure 1A). Presence of the *Dbi* transcript was detected in both mouse and human DRG (Figure 1B). Analysis of human single-nucleus transcriptomic data (37) revealed coclustering of *DBI* with *FABP7*, a validated SGC marker (38) (Supplemental Figure 1A; supplemental material available online with this article; https://doi.org/10.1172/JCI176227DS1). Additionally, mouse RNA-Seq data (39) revealed the highest expression of *Dbi* in the SGCs, moderate expression in the non-myelinating Schwann cells, and low to non-detectable expression in other DRG-resident cells (Supplemental Figure 1B). Immunostaining identified strong abundance of DBI protein in structures enwrapping human (Figure 1C and Supplemental Figure 2, A and B), mouse (Figure 1E and Supplemental Figure 2, C and D), and rat (Figure 1D and Supplemental Figure 2, E and F) sensory neuron somata. DBI immunoreactivity did not overlap with neuronal markers β-tubulin III (mouse, Figure 1F; human, Supplemental Figure 2B), NF200, and peripherin (rat, Supplemental Figure 2F), nor with the macrophage marker IBA-1 and endothelial cell marker CD31 (mouse, Figure 1F). In contrast, strong colocalization with SGC markers FABP7 (mouse, Figure 1F and Supplemental Figure 2C), glutamine synthetase (mouse, Supplemental Figure 2D), and S100B (rat, Supplemental Figure 2E) was observed in DRG sections. Because S100B is also present in Schwann cells (40), we stained the sciatic nerve (Supplemental Figure 2G). A minority of S100B-positive cells in the nerve displayed DBI immunofluorescence, consistent with specific expression in non-myelinating Schwann cells (39) (Supplemental Figure 1B). DBI immunofluorescence colocalized with *Dbi* transcript signal (FISH) in an SGC-like pattern, supporting the specificity of our antibody (Figure 1F). Thus, DBI displays a highly specific expression pattern in mouse, rat, and human DRGs with the highest expression in the SGCs. We also used iDISCO (immunolabeling-enabled 3D imaging of solvent-cleared organs) clearance of the entire rat DRG in combination with light-sheet imaging (3) to visualize the SGC-like 3D pattern of DBI expression in the ganglion (Supplemental Video 1).

### Glial DBI modulates sensitivity to mechanical stimuli.

**Figure 2:**
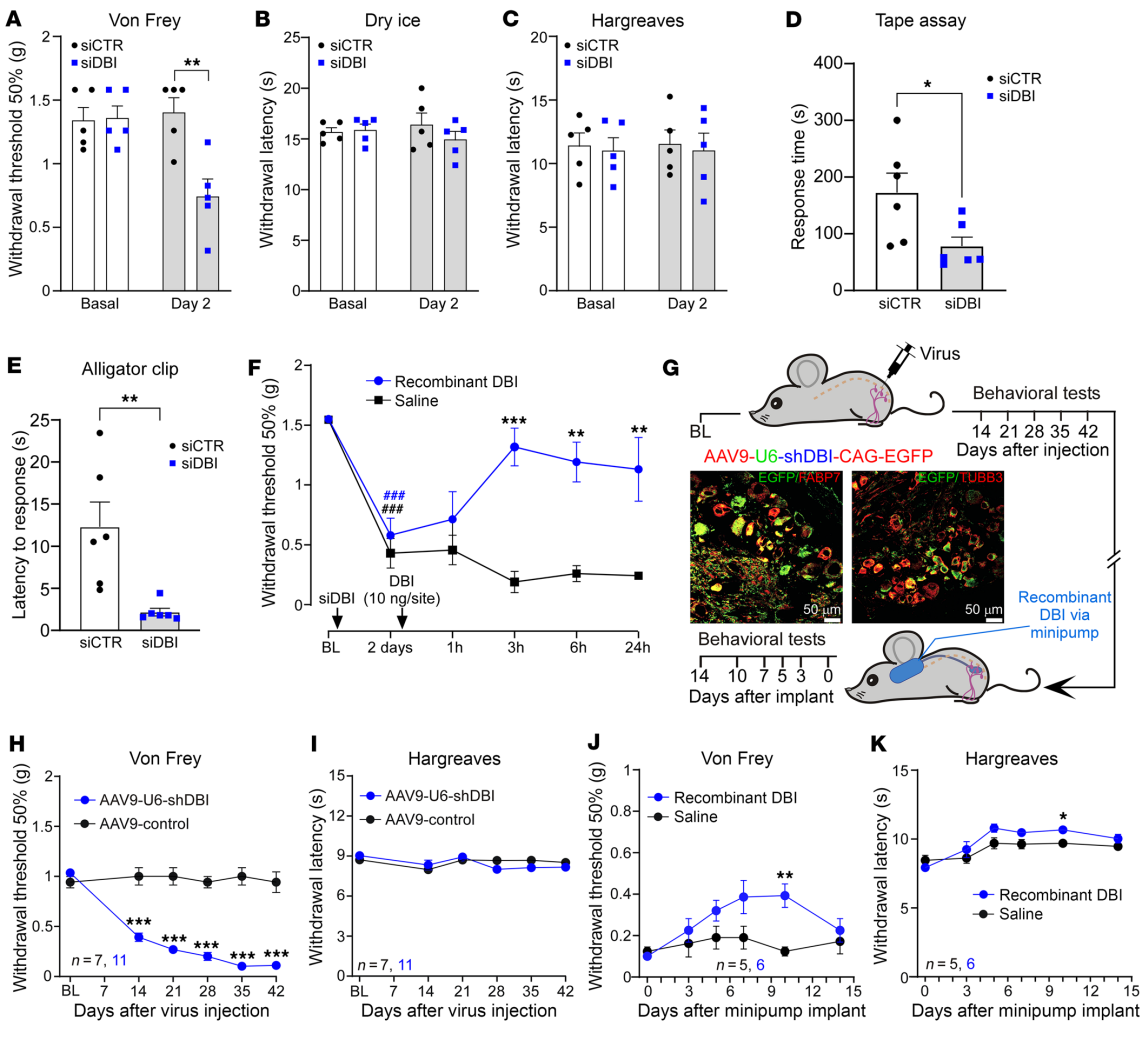
Knockdown of *Dbi* induces mechanical hypersensitivity in mice. (**A**–**E**) siRNA against *Dbi* (or a non-targeting control siRNA) was intrathecally injected (2 mg/site), and 48 hours later the following tests were performed: mechanical sensitivity (von Frey) test (**A**), cold allodynia (dry ice) test (**B**), Hargreaves test (**C**), adhesive removal test (tape assay) (**D**), and alligator clip test (**E**). Bars are mean ± SEM. *P < 0.05; **P < 0.01, significant difference for groups indicated by the connector line (unpaired *t* test). (**F**) Recovery of mechanical hypersensitivity (von Frey test) induced by intrathecal siRNA knockdown of DBI with intrathecal injection of recombinant DBI (10 ng/site). ^###^*P* < 0.001, significant difference from baseline; **P < 0.01, ***P < 0.001, significant difference from time-matched saline group (2-way repeated-measures ANOVA with Šidák’s post hoc test). (**G**) Schematic timeline for the viral DRG gene delivery and osmotic minipump experiment. Inset depicts DRG 8 weeks after injection with AAV9-U6-shDBI-CAG-EGFP virions; this image is included in the extended data set in Supplemental Figure 4A. Scale bars: 50 μm. (**H**) Mechanical sensitivity was monitored with the von Frey test during 42 days after the DRG injection of AAV9-U6-shDBI-CAG-EGFP virions or GFP control virions (1.1 × 10^12^ to 1.2 × 10^12^ viral genomes/mL; 2 μL). ***P < 0.001, significant difference from time-matched control group (2-way repeated-measures ANOVA with Tukey’s post hoc test). (**I**) Similar to **H**, but heat sensitivity was tested with the Hargreaves test. (**J** and **K**) Mechanical (**J**) and heat (**K**) sensitivity was monitored after the implantation of osmotic minipumps delivering recombinant DBI to the DRG (200 μM, 0.5 μL/h; see Methods) to the mice preinjected with the AAV9-U6-shDBI-CAG-EGFP virions. *P < 0.05; **P < 0.01, significant difference from time-matched control group (2-way repeated-measures ANOVA with Tukey’s post hoc test).

Next, we asked what physiological role the SGC-derived DBI might play. Acute knockdown of DBI in the DRG using an intrathecal siRNA in mice revealed a striking phenotype: strong increase in sensitivity to punctate and noxious mechanical stimuli (Figure 2, A and E, and Supplemental Figure 3, B–D and F–I), without a change in thermal sensitivity (either heat or cold; Figure 2, B and C). Sensitivity to innocuous mechanical stimulation (adhesive removal test; Figure 2D) was also increased. Thus, the punctate mechanical withdrawal threshold (von Frey) was more than halved (Figure 2A), responses to noxious pinprick were more than doubled (Supplemental Figure 3, D and H), and the latency to response in the alligator clip test dropped more than 4-fold (Figure 2E and Supplemental Figure 3I). Similar effects were recorded in male and female mice (Supplemental Figure 3, F–I). Sensorimotor coordination (rotarod) was not affected by DBI knockdown (Supplemental Figure 3E). Importantly, mechanical hypersensitivity induced by DBI knockdown was completely rescued by the intrathecal delivery of purified DBI (10 ng/site; Figure 2F).

To produce sustained downregulation of DBI expression in the mouse DRG, we performed intra–L4 DRG injections of anti-*Dbi* shRNA construct incorporated into adeno-associated virions (AAV9-shDBI), which also contained EGFP. Two weeks after injection, the EGFP fluorescence was readily detectable in the DRG (Figure 2G and Supplemental Figure 4A) but not in the spinal cord (Supplemental Figure 5A), and *Dbi* transcript levels in the whole DRG were reduced by approximately 50% (in comparison with the animals receiving EGFP-only control virions; Supplemental Figure 6, A and B). *Dbi* transcript levels in the spinal cord were not affected (Supplemental Figure 5, C and D). Strikingly, AAV9-shDBI (but not AAV9-control) induced strong mechanical hypersensitivity, manifested in dramatic sensitization to mechanical, but not thermal, stimulation (Figure 2, H and I), which became significant at 14 days after viral infection and persisted for the duration of the experiment (42 days after injection). The hypersensitivity was partially alleviated by DRG delivery of recombinant DBI via the implanted minipump (Figure 2, G, J, and K). Notably, approximately 2-week delay is expected for AAV-mediated transgene expression in vivo (41). There was also no change to noxious mechanical sensitivity in the contralateral paw (Supplemental Figure 6, D and E). Sensitivity to innocuous mechanical stimulation (cotton swab test; not shown) was unchanged.

**Figure 3:**
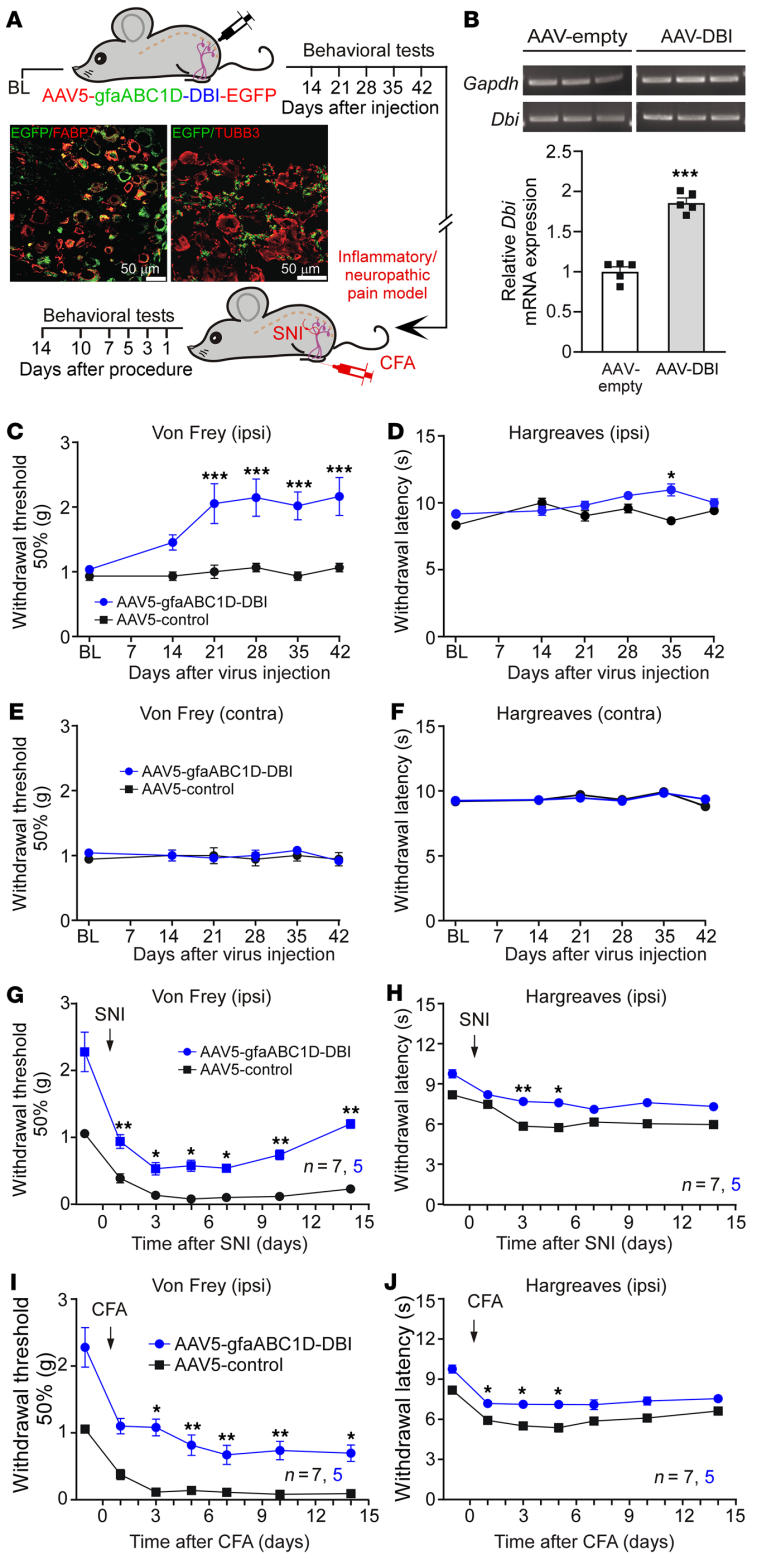
SGC-targeted DBI overexpression reduces mechanical sensitivity in naive mice and suppresses mechanical allodynia in neuropathic and inflammatory pain models. (**A**) Schematic timeline for the viral DRG gene delivery and chronic pain induction experiments. Inset depicts DRG 8 weeks after injection with AAV5-gfaABC1D-DBI-EGFP virions; this image is included in the extended data set in Supplemental Figure 4B. Scale bars: 50 μm. (**B**) Reverse transcription PCR confirmation of *Dbi* overexpression in the DRG. ***P < 0.001, significant difference from control group (unpaired *t* test). (**C** and **D**) Mechanical (von Frey; **C**) and heat (Hargreaves; **D**) sensitivity was monitored on the ipsilateral paws during 42 days after the DRG injection of AAV5-gfaABC1D-DBI-EGFP virions or GFP control virions (1.1 × 10^12^ to 1.2 × 10^12^ viral genomes/mL; 2 μL). *P < 0.05 ***P < 0.001, significant difference from time-matched control group (2-way repeated-measures ANOVA with Tukey’s post hoc test). (**E** and **F**) Similar to **C** and **D**, but the tests were conducted on the contralateral paws. (**G** and **H**) Mechanical (**G**) and heat (**H**) sensitivity was monitored after induction of the spared nerve injury (SNI) model (see Methods) to the mice preinjected with AAV5-gfaABC1D-DBI-EGFP virions. (**I** and **J**) Mechanical (**I**) and heat (**J**) sensitivity was monitored after induction of the complete Freund’s adjuvant (CFA) inflammatory pain model (see Methods) to the mice preinjected with AAV5-gfaABC1D-DBI-EGFP virions. (**G**–**J**) *P < 0.05, **P < 0.01, significant difference from time-matched control group (2-way repeated-measures ANOVA with Tukey’s post hoc test).

Viral constructs used in the above experiments carried general U6 promoter to drive shRNA expression; this would not target SGCs specifically. Hence, in the next experiments, we constructed AAV5 virions with DBI expression under control of the astroglial GFAP promoter (gfaABC1D), which has successfully been used for viral overexpression of genes in SGCs (42). In the first experiment we asked whether SGC-specific DBI overexpression would reduce sensitivity to noxious mechanical stimulation. To this end, we injected AAV5-gfaABC1D-DBI (or EGFP control) into L4 DRG of mice. This resulted in strong overexpression of DBI in the DRG with EGFP fluorescence often displaying a characteristic “ring” pattern (Figure 3, A and B, and Supplemental Figure 4B); expression of DBI in the spinal cord was not affected (Supplemental Figure 5, B–D). Glia-specific DBI overexpression reduced mechanical sensitivity on the ipsilateral side (significant from day 21 after viral injection; Figure 3C) with no contralateral effect (Figure 3E). Thermal sensitivity was minimally affected on the ipsilateral and contralateral sides (Figure 3, D and F).

Next, we tested whether DBI overexpression would alleviate mechanical hypersensitivity in mouse models of chronic inflammatory or neuropathic pain. We injected AAV5-gfaABC1D-DBI (or EGFP control) into L4 DRG of mice 40 days before partial sciatic nerve ligation (spared nerve injury [SNI], a neuropathic pain model; Figure 3, A, G, and H) or hind-paw injection of complete Freund’s adjuvant (CFA; an inflammatory pain model; Figure 3, A, I, and J). Behavioral tests were performed before and after the procedure. Consistent with previous experiments, SGC-specific overexpression of DBI markedly decreased background mechanical sensitivity with no substantial effect on background heat sensitivity. Importantly, mechanical hypersensitivity induced in both SNI and CFA models was significantly attenuated for the duration of either of the experiments (14 days after the procedure; Figure 3, G and I). In the case of SNI, there was a marked recovery of mechanical hypersensitivity, not seen in the control (AAV5-gfaABC1D-EGFP) animals by day 14 after injury (Figure 3G). Interestingly, in both models, there was a slight but significant attenuation of heat hypersensitivity at the initial stages of the experiment (Figure 3, H and J). No significant effects were observed in either model on the contralateral paw (Supplemental Figure 6, F–I).

Taken together, data presented in Figures 2 and 3 reveal that manipulations with DBI abundance in SGCs produce a specific effect on animal sensitivity to noxious and innocuous mechanical stimuli, while other sensory modalities remain minimally affected: reduction in DBI expression results in hypersensitivity, while overexpression of DBI (or exogenous DBI delivery) reduces mechanical sensitivity.

### SGCs release DBI.

**Figure 4:**
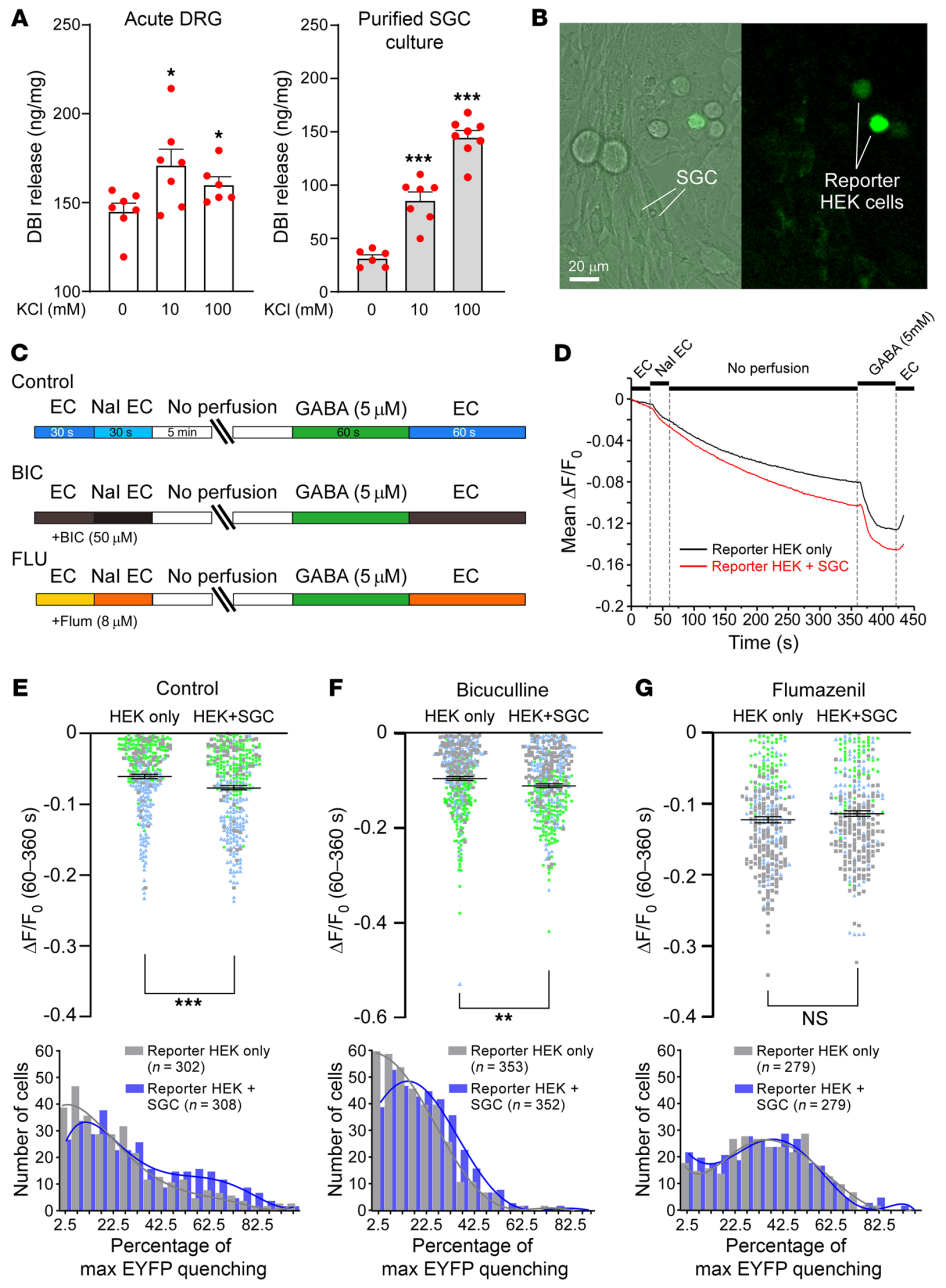
DBI is released by SGCs. (**A**) Detection of DBI by ELISA in extracellular media from acutely extracted DRG (containing all the cell types within the ganglion, “Acute DRG”) and from purified mouse SGC culture (“Purified SGC culture”). Measurements were made after 30 minutes of incubation in control medium and in medium with high extracellular [K^+^] (10 or 100 mM). *P < 0.05; ***P < 0.001, significant difference from 0 mM KCl group (1-way ANOVA with Tukey’s post hoc test). (**B**–**G**) Detection of endozepine release by cultured purified rat SGCs using “reporter” HEK293 cells transfected with α_1_, β_2_, and γ_2_ subunits of GABA_A_ receptors and a halide-sensitive EYFP mutant (H148Q/I152L; EYFP-QL). (**B**) Micrographs depicting coculture of reporter HEK293 cells (green) with purified primary rat SGC culture. Scale bars: 20 μm. (**C**) Schematic of experimental timeline. (**D**) Example of the experiment: Reporter HEK293 cells alone (black line) or in coculture with SGCs (red line) are imaged in the presence of 5 mM extracellular iodide. After introduction of the I^–^-containing solution, the perfusion is stopped for 5 minutes to allow releasable molecules to accumulate. When GABA_A_ receptors are activated, I^–^ enters the cells and produces EYFP-QL fluorescence quenching. GABA (5 μM) is added at the end of the experiment to authenticate fluorescence quenching. (**E**–**G**) Top panels display mean data for EYFP-QL quenching of reporter HEK293 cells only (black symbols) or in coculture with SGCs (blue symbols) in control conditions (**E**) or in the presence of the GABA_A_ receptor blocker bicuculline (50 μM; **F**) or the benzodiazepine antagonist flumazenil (8 μM; **G**). Data from each biological replicate are color-coded. Bottom panels show frequency distribution histograms for the data sets summarized in the corresponding top panels. **P < 0.01, ***P < 0.001, significant difference from the reporter HEK293 cells only (Mann-Whitney test).

DBI lacks a conventional signal peptide and, thus, is not known to be packaged and released via “classic” vesicular secretion (25, 43). Yet astrocytes readily release DBI into the extracellular media (44, 45). Furthermore, release from astrocytes of the DBI cleavage product octadecaneuropeptide DBI_33–50_ (ODN) and likely DBI itself can be strongly induced by depolarization with extracellular potassium (46). Thus, (a) astroglia can release DBI, and (b) such release can potentially be activity dependent. Next, we investigated whether SGCs can also release DBI. We first tested whether DBI can be detected in the extracellular media of cultured mouse DRG cells by ELISA. Indeed, DBI was readily detectable in the conditioned medium of both acutely extracted DRGs and purified SGC cultures (Figure 4A). Additionally, in both cell preparations, DBI release was significantly induced by incubation in the media containing either 10 mM or 100 mM extracellular K^+^, which was consistent with earlier astrocyte experiments (46).

We hypothesized that DBI released from SGCs could act on neuronal GABA_A_ receptors within the DRG to modulate mechanical sensitivity. Thus, we tested whether SGCs can modulate GABA_A_ receptors in a paracrine fashion. To this end, we cocultured purified rat SGCs with “reporter” HEK293 cells overexpressing α_1_/β_2_/γ_2_ GABA_A_ receptor subunits together with the halide-sensitive EYFP mutant (H148Q/I152L; EYFP-QL; Figure 4B). The fluorescence of EYFP-QL is quenched by halide ions, such as iodide. Since GABA_A_ channels are permeable to iodide, EYFP-QL fluorescence quenching by the influx of I^–^ added to the extracellular solution can be used to monitor GABA_A_ channel activation (3, 47). We reasoned that if SGCs can release a GABA_A_ receptor ligand or modulator, we would be able to register it as an EYFP-QL fluorescence quenching when purified SGCs are cocultured with the reporter HEK cells. Live fluorescence recordings were performed in SGC/reporter HEK cocultures (Figure 4, B–D), an extracellular solution containing 5 mM NaI was introduced into the perfusion chamber, and perfusion was stopped for 5 minutes to allow release of glial factors to take place. At the end of the experiment, perfusion was recommenced, and GABA (5 mM) was applied as a positive control (Figure 4, C and D). EYFP-QL fluorescence quenching over the 5-minute stop-flow period was quantified, and the experiment was repeated in the presence of the GABA_A_ receptor blocker bicuculline (50 μM) or the benzodiazepine antagonist flumazenil (8 μM). The EYFP-QL fluorescence quenching was significantly higher in the presence of SGCs, as compared with reporter HEK cell monoculture (Figure 4, D and E; this is also seen as a rightward shift in the frequency distribution shown in Figure 4E, bottom). Interestingly, this SGC-induced quenching increment was blocked by flumazenil (which antagonizes benzodiazepine but not GABA binding; Figure 4G) but not by bicuculline (which antagonizes GABA but not benzodiazepine binding; Figure 4F). These data support the hypothesis that SGCs release a substance activating or positively modulating GABA_A_ receptors at the benzodiazepine binding site.

### DBI acts as an unconventional agonist and a PAM at GABA_A_ receptors.

**Figure 5:**
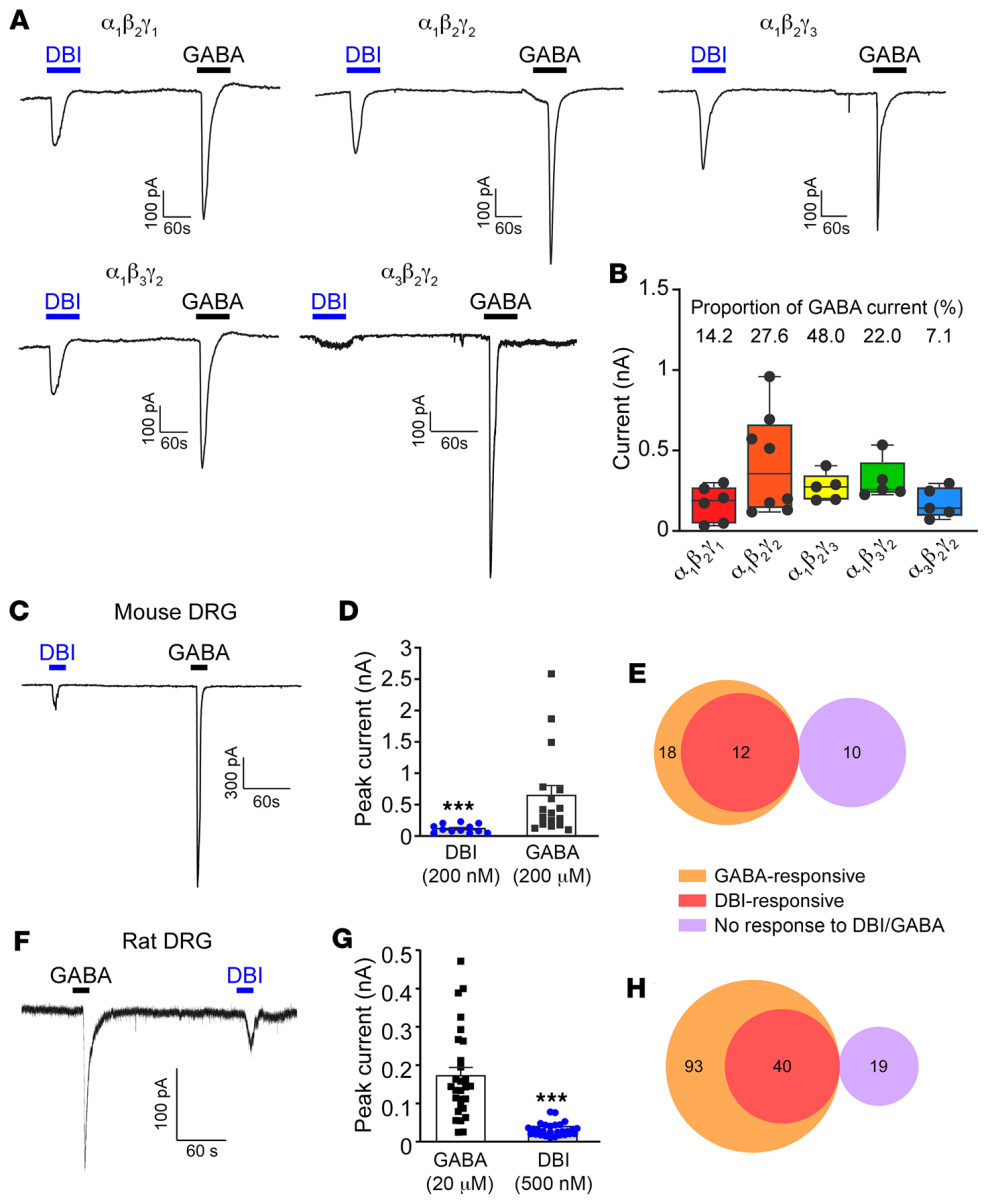
DBI is an unconventional agonist at heterologous and endogenous GABA_A_ receptors. (**A**) Example current traces of whole-cell patch clamp recordings from HEK293 cells heterologously transfected with various combinations (as indicated) of mouse α, β, and γ GABA_A_ receptor subunits. Recombinant purified DBI (200 nM) or GABA (200 μM) was applied via localized perfusion system during periods depicted by the horizontal bars. (**B**) Summarized amplitudes of DBI-induced inward currents for experiments exemplified in **A**. DBI-induced current amplitudes, expressed as a proportion of the GABA_A_ current recorded from the same cell, are summarized above the bars. (**C**–**H**) DBI- and GABA-induced currents recorded from cultured mouse (**C**–**E**) and rat (**F**–**H**) DRG neurons. (**C** and **F**) Example whole-cell patch clamp recordings. (**D** and **G**) Summarized DBI and GABA current amplitudes for experiments exemplified in **C** and **F**. ***P < 0.001, significant difference between the DBI and GABA current amplitudes (unpaired *t* test). (**E** and **H**) Venn diagrams depicting relationships between the GABA-responsive, DBI-responsive, and non-responsive DRG neurons from mouse (**E**) and rat (**H**) DRG cultures.

We next asked whether purified recombinant DBI can modulate GABA_A_ receptors. First, we transfected HEK293 cells with several combinations of α, β, and γ GABA_A_ receptor subunits and tested whether these can be activated by recombinant DBI (Figure 5, A and B) using patch clamp recording. In all combinations tested (α_1_β_2_γ_1_, α_1_β_2_γ_2_, α_1_β_2_γ_3_, α_1_β_3_γ_2_, and α_3_β_2_γ_2_), DBI induced measurable inward currents kinetically similar to the responses to GABA. Responses to 200 nM DBI ranged from approximately 10% to approximately 50% of the 200 μM GABA response amplitude (Figure 5B) and were somewhat higher for GABA_A_ receptors containing α_1_ and γ_3_ or γ_2_ subunits, as compared with those containing α_3_ or γ_1_ (Figure 5B). There are multiple benzodiazepine binding sites within GABA_A_ channels (48); however, the “classical” high-affinity binding site is formed at the interface between α_1_ and γ_2_ subunits (48, 49). A histidine at position 101 in the α_1_ subunit (50) and phenylalanine at position 77 in γ_2_ were shown to be critical for high-affinity benzodiazepine binding (51). Introduction of these mutations in the α_1_β_2_γ_2_ GABA_A_ channels significantly reduced DBI-induced current amplitudes with only negligible currents recorded from the α_1_(H101R)β_2_γ_2_(F77I) channels (Supplemental Figure 7, A–F). Introduction of homologous H126R mutation into the α_3_ subunit effectively wiped out the DBI responses (Supplemental Figure 7, G–L).

**Figure 6:**
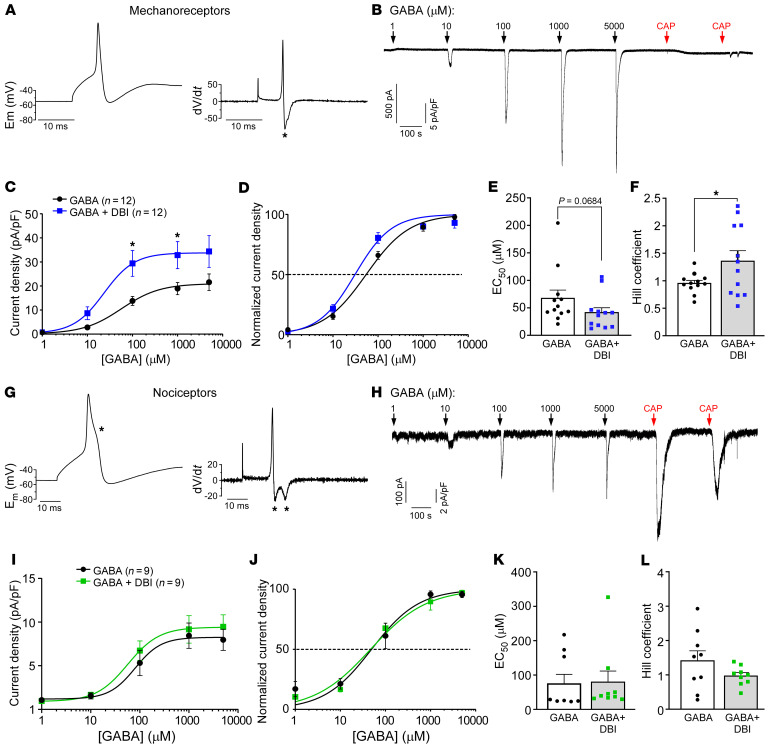
DBI is a positive allosteric modulator at GABA_A_ receptors in presumed mechanosensitive DRG neurons. Shown are the results of the perforated patch recordings from cultured rat DRG neurons. (**A** and **B**) Determinants of a putative mechanosensitive DRG neuron: narrow action potential with a single minimum in the first derivative (**A**) and no response to 1 μM capsaicin. Single minimum in the first derivative is identified with an asterisk. An example of concentration dependence of responses to GABA (1–5,000 μM) is shown in **B**. (**C** and **D**) Absolute (**C**) and normalized (**D**) concentration dependence of GABA currents (quantified as current densities) in the absence (black) and presence (blue) of 500 nM DBI. *P < 0.01, significant difference from concentration-matched control group (2-way repeated-measures ANOVA with Bonferroni’s post hoc test). (**E** and **F**) Summaries of the EC_50_ (**E**) and Hill coefficient (**F**) for experiments exemplified in **B**–**D**. *P < 0.05, significant difference in the Hill coefficient between the indicated groups (unpaired *t* test). (**G** and **H**) Determinants of a putative polymodal nociceptor: broad action potential with 2 minima in the first derivative (**G**) and robust response to 1 μM capsaicin. In **G** (left panel), a “hump” in the action potential waveform is identified with an asterisk. The two minima in the first derivative (right panel) are also identified with asterisks. An example of concentration dependence of responses to GABA (1–5,000 μM) is shown in **H**. (**I**–**L**) Similar to **C**–**F** but for the putative nociceptors.

When applied to cultured DRG neurons, DBI (200–500 nM) also induced GABA-like inward currents in mouse (Figure 5, C–E) and rat (Figure 5, F–H) DRG neurons. The amplitudes of the DBI-induced currents were in the range of 20%–25% of GABA responses; 67% of mouse and 43% of rat GABA-responsive DRG neurons also responded to DBI, while no GABA-negative neurons responded to DBI in either species (Figure 5, E and H). We then tested whether DBI can modulate GABA responses of rat DRG neurons. To characterize sensory modality of recorded neurons, we used an approach described by Smith and Lewin, whereby C-type nociceptors can be electrophysiologically identified by wide action potential with an inflection on the repolarization phase (manifesting as an additional minimum in the first derivative of the spike [dV/dt]), while larger-diameter, presumed Aβ mechanosensitive neurons have narrower spikes with only one minimum in the first derivative (52). We also used sensitivity to 1 μM capsaicin to identify TRPV1-positive nociceptors (Figure 6, A, B, G, and H). Interestingly, in larger, presumed mechanosensitive neurons (one minimum, capsaicin-negative; Figure 6, A–F), DBI (500 nM) produced strong potentiation of GABA responses at all concentrations, a significant increase of the Hill coefficient, and a trend toward increased affinity (EC_50-control_ = 67.6 ± 14.7 μM, EC_50-DBI_ = 41.2 ± 9.0 μM; *P* = 0.0684). On the other hand, DBI had no significant effects on either concentration dependence or maximal response to GABA in presumed small nociceptors (two minima, capsaicin-positive; Figure 6, G–L). These data suggested that DBI acts as a PAM at GABA_A_ receptors in presumed larger-diameter neurons responsive to mechanical stimuli but not in TRPV1-positive nociceptors. This unexpected result hinted toward a potential explanation for why manipulations with DBI expression or abundance at the DRG affected mechanical but not thermal sensitivity in vivo.

### Specificity of DBI signaling toward mechanosensitive fibers.

**Figure 7:**
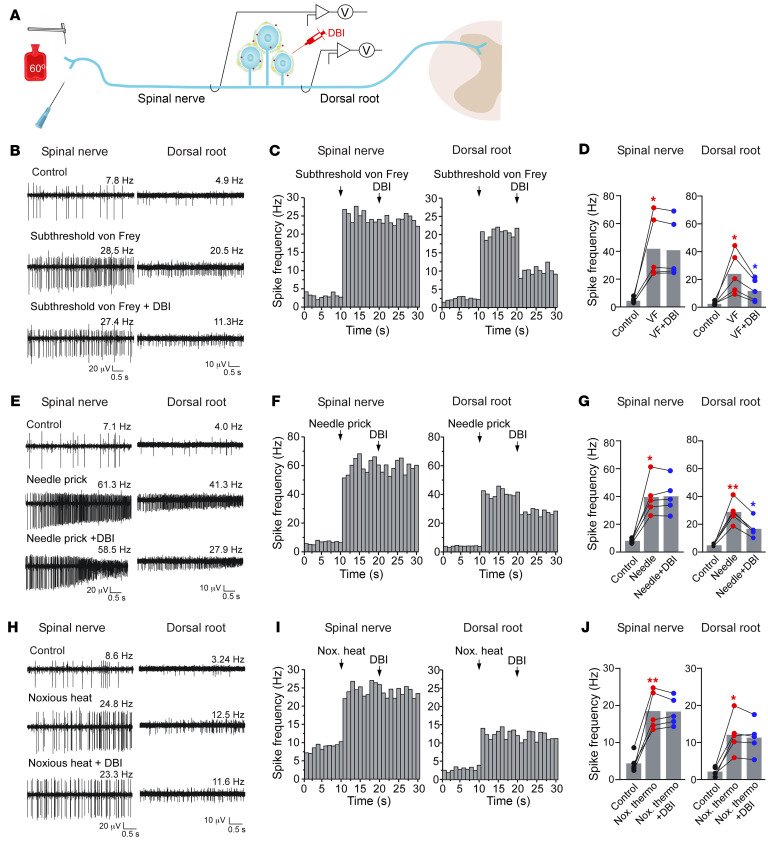
DBI-induced filtering of peripheral nerve activity at the DRG. (**A**) Schematic of the recording method. Experiments were performed in mice. Spinal nerve and dorsal root were surgically exposed and then individually suspended on fine hook electrodes; DRG was exposed to direct drug application. (**B**) Subthreshold mechanical stimulus (von Frey filament, 0.6 g) increased firing frequency in both spinal nerve and dorsal root branches of the nerve (middle traces, as compared with basal activity shown in the top traces). Application of DBI (200 μM, 2 μL) to the DRG reduced mechanical stimulus–induced firing frequency in the dorsal root but not in the spinal nerve (bottom traces). (**C**) Histogram of firing frequencies of spinal nerve and dorsal root branches of the nerve from experiment exemplified in **B**. (**D**) Summary of experiments exemplified in **B**. (**E**–**G**) Experiment similar to that shown in **B**–**D**, but a noxious mechanical stimulus (needle prick) was used. (**H**–**J**) Experiment similar to those shown above, but a noxious thermal stimulus (hot water, 60°C) was used instead of mechanical stimuli. One-way repeated-measures ANOVA with Tukey’s post hoc test. (**D**, **G**, and **J**) *P < 0.05, **P < 0.01, significant difference from previous group.

We recently reported a method for in vivo electrophysiological recording of spike filtering at the DRG using simultaneous extracellular recordings from the peripheral and central branches of the L5 spinal nerve of a rat (3). This preparation allows synchronous measurement of spikes in the nerve branch before and after the DRG and, thus, allows quantification of ganglionic “filtering.” We used this method to assess how DRG application of DBI would affect filtering of spikes induced by different types of stimuli applied to the paw (Figure 7A). Consistent with our behavioral experiments, DBI injected into the L4 DRG of a mouse significantly increased filtering of spikes induced by mechanical stimulation of the paw, both innocuous (sub-threshold von Frey filament; Figure 7, B–D) and noxious (needle prick; Figure 7, E–J). On the other hand, filtering of the spikes induced by noxious thermal stimulus (60°C) was not affected (Figure 7, H–J).

**Figure 8:**
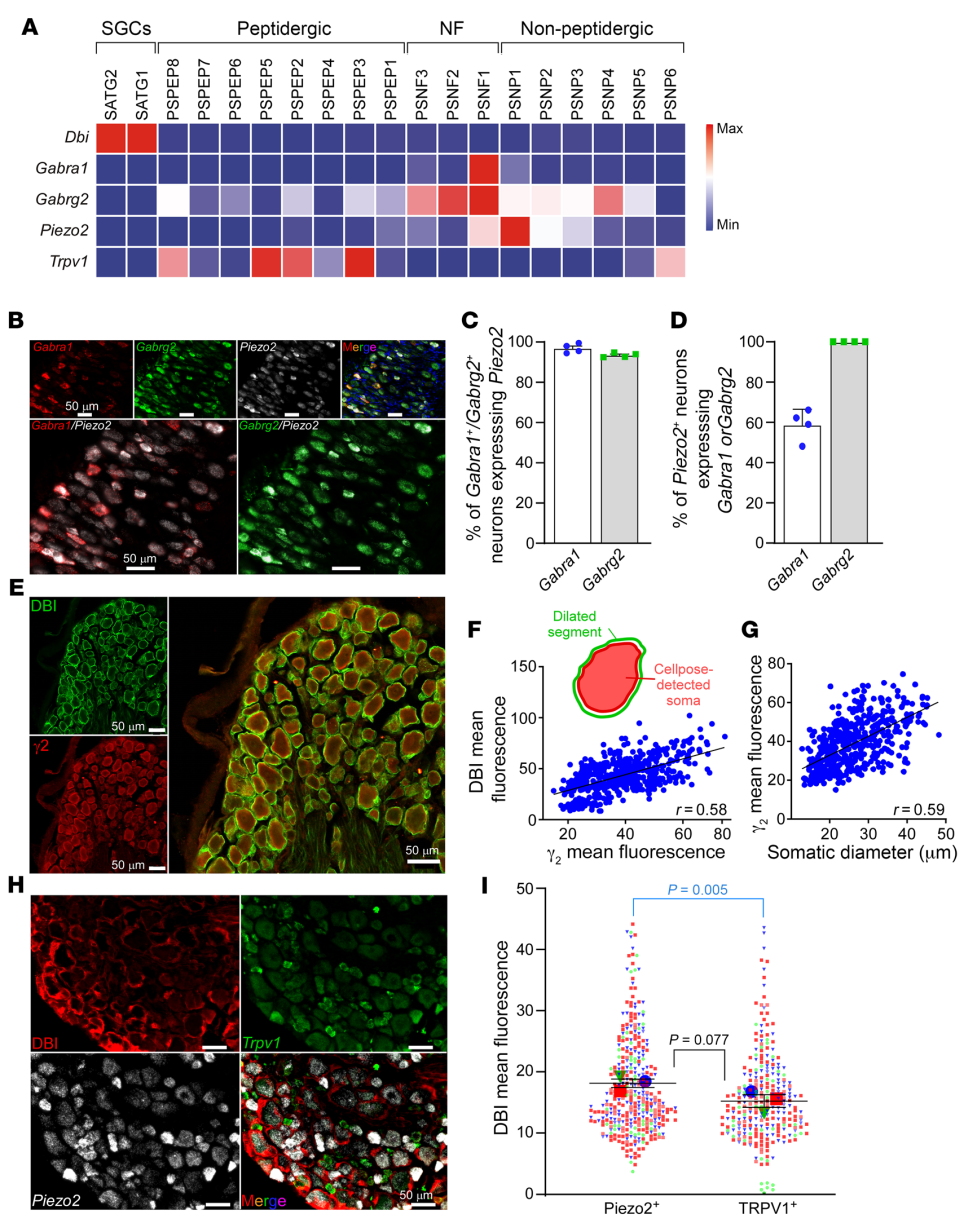
Mechanosensitive neurons are specifically coupled to DBI-expressing glia. (**A**) Heatmap for expression of *DBI*, *Gabra1*, *Gabrg2*, *Piezo2*, and *TRPV1* in different populations of mouse DRG neurons and glia; data from ref. 53. (**B**–**D**) FISH analysis of *Gabra1*, *Gabrg2*, and *Piezo2* mRNA expression in mouse DRG. Example stainings are shown in **B**. Scale bars: 50 μm. Proportions of *Gabra1^+^* and *Gabrg2^+^* neurons that were also positive for *Piezo2* are analyzed in **C**; proportions of *Piezo2*^+^ neurons that were also positive for either *Gabra1* or *Gabrg2* are analyzed in **D**. (**E**–**G**) Colocalization of γ_2_ GABA_A_ and DBI immunofluorescence in rat DRG. Example stainings are shown in **E**. Scale bars: 50 μm. (**F**) Cell bodies of the γ_2_-positive DRG neurons were auto-detected with Cellpose, and the automatically detected segments were radially dilated by 10 and shrunk by 3 pixels. DBI immunofluorescence was analyzed in the “doughnut” space between the contracted and dilated segments (schematized in the inset; see Methods). Plots show correlation between the somatic γ_2_ mean fluorescence and “doughnut” DBI mean fluorescence. (**G**) Correlation between the somatic diameter of the DRG neurons and DBI integrated density. (**H** and **I**) DBI immunofluorescence and FISH analysis of *Trpv1* and *Piezo2* mRNA expression in mouse DRG. Example stainings are shown in **H**. Scale bars: 50 μm. In **I**, a similar approach to that shown in **F** is shown: cell bodies of *Trpv1^+^* or *Piezo2^+^* neurons were auto-detected, and the DBI immunofluorescence in the dilated segments was measured (see Methods) and compared between the *Trpv1^+^* and *Piezo2^+^* neurons. Data from each biological replicate are color-coded; means from each replicate are shown in large symbols. *P* = 0.077 is for comparison of means per replicate; *P* = 0.005 is for comparison of means of individual values (unpaired *t* test).

The majority of sensory neurons express functional GABA_A_ receptors (Figure 5, E and H) (2); thus the question arises: why is inhibitory action of DBI specific to mechanosensitive neurons? Sensitivity of GABA_A_ receptors to benzodiazepines strongly depends on the subunit composition of the channel complex, and it is strongest for the channels containing α_1_ and γ_2_ subunits (reviewed in ref. 48). Bioinformatic analysis of the RNA-Seq data of mouse DRG neurons (53) revealed that transcripts of these subunits (and particularly α_1_) are mostly found in A-type, Piezo2-expressing neurons and not in C-type, TRPV1-expressing ones (Figure 8A). FISH experiments on mouse DRGs (Figure 8B) confirmed a high degree of coexpression between *Piezo2*, α_1_ (*Gabra1*), and γ_2_ (*Gabrg2*) transcripts: *Piezo2* was found in 96% of *Gabra1^+^* and 93% of *Gabrg2^+^* neurons; 100% of *Piezo2^+^* neurons contained *Gabrg2* and 55% contained *Gabra1* (Figure 8, C and D).

Double staining against γ_2_ and DBI revealed strong positive correlation between the neuronal γ_2_ immunofluorescence intensity and DBI immunofluorescence intensity in the neuron-wrapping SGCs (Figure 8, E and F; see Methods for quantification protocol). For both α_1_ and γ_2_ proteins, there was a strong positive correlation between the expression level and cell diameter (Figure 8G and Supplemental Figure 8, A and B), confirming preferential expression in larger, A-type fibers. This result suggested that larger, *Gabrg2^+^* neurons have more DBI in their glial wrapping as compared with smaller, *Gabrg2^–^* neurons. We expect similar relationships to exist between α_1_ expression and the abundance of DBI in the surrounding SGCs, since *Gabra1* expression is restricted to *Gabrg2^+^* and *Piezo2^+^* neurons (Figure 8A; see below).

Using a combination of DBI immunofluorescence and RNAscope detection of *Piezo2* and *Trpv1*, we were able to compare the DBI levels in SGCs surrounding *Piezo2^+^* and *Trpv1^+^* neurons. The DBI abundance showed a bias toward the *Piezo2^+^* neurons (Figure 8, H and I). Thus, it appears there is a twofold mechanism ensuring preferential coupling of DBI signaling to mechanosensitive neurons: (a) these preferentially express GABA_A_ receptor subunits necessary for the assembly of high-affinity benzodiazepine binding site, which, in turn, is required for potentiation of GABA_A_ activity by DBI; (b) the SGCs around these neurons have higher levels of DBI expression as compared with those wrapping smaller-diameter C-type neurons.

### DBI modulates mechanosensitivity acting at the GABA_A_ receptors, not on its other binding partners.

Although identified as a GABA_A_ binding protein, DBI has other binding partners, including acyl-CoA and the mitochondrial DBI receptor TSPO (translocator protein) (25, 54). Hence, our next experiments were designed to confirm the role of the GABA system and assess the potential contribution of the other DBI targets in its action within the DRG.

Importantly, siRNA knockdown of either *Gabra1* or *Gabrg2* by intrathecal siRNA injection (mouse) recapitulated DBI knockdown phenotype with strong mechanical hypersensitivity (von Frey) but nearly normal sensitivity to heat (Hargreaves test) or cold (dry ice test) and no discernible motor deficits (rotarod) (Supplemental Figure 9, A–G). Moreover, in mice, the DBI knockdown–induced mechanical hypersensitivity was almost completely reversed by systemic administration of the GABA_A_ agonist isoguvacine (2 mg/kg, i.p.; Supplemental Figure 10A). Isoguvacine is a peripherally restricted agonist that was shown to target peripheral DRG neurons when given systemically (55); hence, its efficacy to recover DBI knockdown–induced mechanical hypersensitivity reinforces the notion that DBI acts via the GABA system.

Next, we tested whether the anti-nociceptive effect of DBI can be perturbed by disabling of the acyl-CoA binding site of DBI. To this end, we performed chronic pain model experiments in which either SNI (Supplemental Figure 10, B–D) or CFA (Supplemental Figure 10, B, E, and F) models were established on mice pre-implanted with osmotic minipumps for DRG drug delivery (2). In these experiments, DBI alleviated mechanical but not thermal hypersensitivity in both SNI and CFA models, as did the DBI(K33A) mutant; this mutation dramatically reduces DBI’s affinity to acyl-CoA, which, in turn, is necessary for mitochondrial effects of DBI binding to TSPO (25, 56). Furthermore, we tested whether the anti-nociceptive effect of DBI can be antagonized by coinjection with either the TSPO antagonist PK11195 or the benzodiazepine antagonist flumazenil. We repeated chronic pain model experiments, in which either SNI or CFA models were established on mice pre-implanted with a DRG cannula (Supplemental Figure 10G). In these experiments, DBI coapplied with PK11195 still produced strong anti-nociceptive effect in von Frey experiments, while DBI coapplied with flumazenil did not produce such action. These data further support our conclusion that the primary mechanism of DBI action within the DRG is indeed via the GABA system.

## Discussion

We present here a case for somatic SGC-to-neuron communication within the dorsal root ganglia by the glial endozepine peptide DBI. The peptide is produced and released by the glial cells; upon release, DBI binds to the GABA_A_ receptors on the sensory neurons, presumably at the somatic/perisomatic areas, and potentiates GABA_A_-mediated inhibition. In vivo, this action manifests as reduction in sensitivity to mechanical stimulation (in noxious and innocuous range). Both global and SGC-specific DBI knockdown in the DRG results in marked mechanical hypersensitivity. This hypersensitivity can be alleviated by an intrathecal injection of purified recombinant DBI. On the other hand, DBI overexpression (or minipump delivery into the DRG) results in reduction in basal mechanical sensitivity and alleviates mechanical hypersensitivity induced by nerve injury or chronic inflammation.

Remarkably, while effective at modulating mechanical sensitivity, DBI mostly spares thermosensitivity. Thus, sensitivity to cold and heat was largely unaffected by either knockdown, overexpression, or exogenous applications of DBI in our experiments (some minor effects on heat sensitivity were observed in some settings). In searching for an explanation for this unexpected specificity, we discovered that Piezo2-positive mechanosensitive DRG neurons are much more likely to express a combination of α_1_ and γ_2_ GABA_A_ receptor subunits necessary for the assembly of high-affinity benzodiazepine binding site (48, 49) than any other type of DRG neuron (Figure 8). Additionally, immunohistochemical and RNA labeling revealed that satellite glial cells surrounding these Piezo2-positive, presumed mechanosensitive neurons express much higher amounts of DBI as compared with smaller-diameter neurons, such as TRPV1-positive nociceptors. Thus, there is a twofold mechanism ensuring specific targeting of neurons responsive to mechanical stimuli by DBI.

On the cellular level, DBI acts as an unconventional GABA_A_ receptor agonist and, in addition, as a PAM at those receptors that contain the high-affinity benzodiazepine binding site (i.e., those containing α_1_ and γ_2_ subunits). Thus, both in a heterologous expression system and in native DRG neurons, DBI (in the 100 nM range) induces GABA-like currents of approximately one-quarter of the maximal GABA current amplitude (Figures 5 and 6), which are abolished by mutations within the high-affinity benzodiazepine binding site (Supplemental Figure 7). Additionally, DBI strongly potentiates the responses of high-affinity GABA_A_ receptors to GABA (Figure 6). It was challenging to establish precise concentration dependence of DBI effect on GABA_A_ currents because of stability issues and aggregation of purified peptide, but empirically, we established that raising the concentration of DBI above 200–500 nM did not increase GABA_A_ current responses in the patch clamp experiments. Behavioral experiments suggest that neither TSPO nor acyl-CoA contributes substantially to the sensory manifestations of the DBI action within the DRG (Supplemental Figure 10). Finally, mechanical hypersensitivity produced by DBI knockdown can be mimicked by the knockdown of α_1_ or γ_2_ GABA_A_ receptor subunits in the DRG and alleviated by a peripherally restricted GABA_A_ agonist, isoguvacine (Supplemental Figure 10). All this evidence strongly suggests that it is indeed the benzodiazepine-like activity of DBI that is necessary for its anti-nociceptive action.

Recent findings suggest that the intrinsic GABAergic system present within the DRG acts as a “peripheral gate” within the somatosensory system (2, 3, 5). Importantly, direct DRG application of GABA reuptake inhibitor produces strong anti-nociceptive effects (2, 16), while DRG injections of GABA_A_ receptor antagonists exacerbated peripherally induced nociception (2) and reduced tonic spike filtering at the DRG in vivo (3). These experiments imply the presence of GABA tone within the DRG. Intriguingly, viral overexpression of the GABA-producing enzyme glutamic acid decarboxylase (GAD65) in trigeminal SGCs produced strong trigeminal analgesia (57). Hence, the most straightforward mechanistic explanation for the anti-nociceptive effect of DBI is its release from the SGCs and paracrine action on the GABA_A_ receptors on the sensory neurons, whereby it produces a benzodiazepine-like amplification of GABA tone for those neurons expressing the high-benzodiazepine-affinity GABA_A_ receptors, such as Piezo2-positive mechanosensitive neurons. One potential issue with this hypothesis is that according to our recent data (3), it is C-type, not A-type, fibers that are the most sensitive to GABA_A_-mediated inhibition. C-fibers have a particularly short stem axon (over 3-fold shorter than in A-fibers), which ensures good electrotonic coupling between the soma and T-junction in C-fibers with much poorer coupling in A-type fibers. Hence, somatically released GABA would have higher influence over T-junctional spike filtering in C-type as compared with A-type fibers (3). How then could DBI target mechanosensitive, mostly A-type, fibers? One hypothesis is that the PAM action of DBI on the mechanosensitive A-type neurons may bring them within the range of susceptibility to the GABA tone. An additional hypothetical mechanism could involve the actions of DBI at the GABA_A_ receptors at or around the mechanoreceptive fiber T-junctions, making stem length less relevant. There could be additional biophysical or other reasons that are yet to be understood.

An important finding in our study relates to the anti-nociceptive signaling of SGCs. While numerous studies have documented the proalgesic action of SGCs in the DRG, such as cross-excitation of neighboring neurons (58, 59) and sensitizing action through the release of proinflammatory cytokines and fractalkine when activated (reviewed in refs. 19, 21), other studies highlight how SGCs also help maintain homeostasis and control chronic pain. For example, SGCs were shown to maintain tonic inhibition of the expression of excitatory P2X3 receptors in DRG neurons (60). Consistent with our results, recent research also revealed that direct chemogenetic activation of SGCs in vivo can produce an analgesic effect in both acute and chronic inflammation pain models (61). Furthermore, our recent studies indicate that G protein–coupled receptor and metalloproteinase signaling in SGCs presents promising therapeutic targets for neuropathy and chronic pain management (62, 63). Thus, while SGCs have historically been studied for their proalgesic functions, our work and other new studies highlight their anti-nociceptive signaling and ability to control homeostasis and chronic pain.

The mechanism(s) of DBI release by astroglial (or other) cells is poorly understood. DBI (and its precursor peptide) lacks classical signal peptide (43), and apparently it is not packaged into secretory vesicles (30). Yet astroglia actively releases both DBI and its cleavage product, ODN (44, 64, 65). Possible mechanisms for DBI release/secretion currently being tested include efflux through the membrane pores, ATP-dependent excretion through the ABC transporters, or release via autophagy-associated vesicles (reviewed in ref. 25). We can readily detect DBI by ELISA in the extracellular media from the full DRG cultures, as well as from the purified SGCs (Figure 4A). Additionally, release by SGCs of a factor acting on the benzodiazepine binding site of GABA_A_ receptors was detected by our “sniffing” imaging approach (Figure 4, C–G). Importantly, as in the case of astrocytes (46), DBI release from the SGCs can be induced by high extracellular K^+^ (Figure 4A), a phenomenon possibly related to the transmembrane transport of molecules associated with K^+^ buffering function of the SGCs in the ganglia (23). The latter finding suggests that DBI release from glia can be stimulated during periods of high firing rates of the DRG neurons, when these actively extrude K^+^ ions via repolarizing voltage-gated K^+^ channels. This could provide an anti-nociceptive dampening mechanism for pain induced by noxious mechanical stimulation.

We also show high abundance of DBI in the human DRGs, both adult and embryonic (Figure 1 and Supplemental Figure 2). Developmental differences in the pharmacological profile of GABA_A_ receptors in human DRGs have been reported, including loss of bicuculline sensitivity in the adult DRG (66). Importantly, both embryonic and adult human DRG GABA_A_ receptors are sensitive to benzodiazepines (66); hence we hypothesize that anti-nociceptive action of DBI is present in human DRGs throughout development. Our data reveal that overexpression or exogenous delivery of DBI to the DRG can alleviate mechanical allodynia in both neuropathic and inflammatory rodent pain models (Figure 3 and Supplemental Figure 10). Together with the peripheral analgesic efficacy of isoguvacine (Supplemental Figure 10), these results uncover a tempting possibility for therapeutic targeting of the GABAergic system within the DRG with peripherally restricted modulators or biologics, opening potential new avenues for pain relief.

## Methods

### Sex as a biological variable

Our study examined male and female animals, and similar findings are reported for both sexes.

### Cell cultures and transfection

DRG cultures from adult male C57BL/6 mice or 5- to 12-day-old Wistar rats were prepared by enzymatic dissociation (collagenase/dispase), as described previously (7). Cultured neurons were used for patch clamp recording within 48 hours.

Purified SGC cultures were prepared from Wistar rats (5–12 days old) or C57BL/6 mice (adult) using the method validated and described in detail by us and others (mouse, refs. 63, 67; rat, refs. 68, 69). Briefly, DRGs from all spinal segments were collected into HBSS (Gibco, UK) supplemented with penicillin 100 U/mL and streptomycin 100 μg/mL and subsequently treated with papain 20 U/mL (all from Sigma-Aldrich) for 30 minutes at 37°C. After 2 washes in HBSS, ganglia were treated for 30 minutes at 37°C with collagenase II (1.5 mg/mL; Sigma-Aldrich). The enzymatic digestion was stopped by washing with 1 mL of DMEM low glucose supplemented with 10% fetal bovine serum, 1% penicillin/streptomycin, and 1% amphotericin B (all from Gibco, UK). Ganglia were triturated in 500 μL of DMEM low glucose by manual pipetting. After the addition of another 5.5 mL of DMEM low glucose, the cell suspension was filtered with 40 μm and then 10 μm cell strainers (Fisher Scientific, UK), poured into 2 uncoated T-25 flasks (Sarstedt), and then placed in a humidified 5% CO_2_ incubator at 37°C. After 3–4 hours, SGCs adhered to the bottom of the flask, and the floating neurons were removed via addition of fresh warm media. Subsequently, DMEM low glucose with supplements was changed after 24 hours and then every second day until cell confluence. At 11 days in vitro, SGCs were rinsed with warm PBS and passaged on 10 mm coverslips (at approximately 60% confluence) in a 24-well plate using 0.25% trypsin-EDTA (Gibco).

HEK293 cells (ATCC or Kunming Cell Bank) were grown in DMEM (Gibco) containing penicillin (100 U/mL; Sigma-Aldrich), streptomycin (100 μg/mL; Sigma-Aldrich), and 10% fetal calf serum (Sigma-Aldrich). HEK293 cells were then plated onto 35 mm glass coverslips precoated with poly-d-lysine (100 g/mL) and transfected with 300 ng of each plasmid using FuGene transfection reagent (Promega) or Lipofectamine 2000 (Thermo Fisher Scientific), according to the manufacturer’s instructions.

### Immunofluorescence

Standard immunohistochemical protocols were used. In general, mice or rats were deeply anesthetized with isoflurane or sodium pentobarbital and transcardially perfused with PBS followed by 4% paraformaldehyde (PFA). Lumbar DRGs were isolated and postfixed in PFA for 1–2 hours before incubation overnight in a 30% sucrose solution. Tissues were then embedded in OCT medium (Tissue-Tek) and cryosectioned at a thickness of 14 μm or were embedded in paraffin and then sectioned at 2–3 μm thickness. Tissue sections were initially washed with PBS followed by blocking with PBS-based solutions containing 1% BSA and/or 10% goat serum and 0.2%–0.3% Triton X-100 for 30–60 minutes. After blocking, tissue sections were incubated overnight with the primary antibodies (listed in Supplemental Table 1). The following day, sections were washed with PBS and incubated for 1 hour at room temperature with the secondary antibodies (Supplemental Table 1). Images were acquired using a Keyence BZ-X800 or Zeiss LSM 700/880 confocal microscope.

Adult human DRGs were blocked and fixed overnight at 4°C in 4% PFA in PBS (pH 7.4) and incubated in 30% sucrose overnight at 4°C. Tissue was embedded in Tissue Plus (Fisher Healthcare), cryosectioned at 14 μm, and stored at −80°C. The cryosectioned tissue sections were washed with PBS followed by incubation in blocking solution (2% of BSA in PBS) for 1 hour and then incubated overnight at 4°C with an anti-DBI antibody (rabbit, 1:1,000; Abcam). Sections were then incubated for 1 hour at room temperature with anti-rabbit Alexa Fluor 546 (1:1,000; Thermo Fisher Scientific). Images were acquired using the Keyence BZ-X800.

Human fetal DRGs were dissected and embedded in paraffin. The sections were deparaffinized in xylene and rehydrated with graded ethanol after being sliced into 2 to 3 μm tissue sections. Antigen retrieval was performed by autoclaving of the samples for 10 minutes at 121°C in a sodium citrate buffer (pH 6.0). Sections were blocked with 10% normal goat serum for 30 minutes at 37°C, washed with PBS twice, and incubated with primary antibodies overnight at 4°C, followed by 2-hour labeling with secondary antibody at room temperature.

### Fluorescence in situ hybridization

DRG harvesting, fixing, and sectioning were performed similarly to immunofluorescence labeling. RNAscope was performed following the manufacturer’s instructions using the RNAscope Multiplex Fluorescent Reagent Kit v2, 323110, or Multiplex Fluorescent Assay, 320850 (Advanced Cell Diagnostics). Probes are listed in Supplemental Table 1. For some experiments, RNAscope signals were combined with immunofluorescence, which was carried out as described above. Images were acquired using Keyence BZ-X810, Leica Stellaris 8, or Zeiss LSM 700 confocal microscopes; at least 3 sections from each animal were used for data analysis. Cells with more than 5 puncta were classified as positive for specific mRNA expression.

### Fluorescence colocalization analysis

For quantification of DBI wrapping, the following approach was used. Regions of interest (ROIs) of DRG neuron cell bodies were detected based on the somatic immunofluorescence (e.g., γ_2_ GABA_A_ subunit) or FISH (e.g., *Trpv1*) signal with the software Cellpose (70). The masks obtained were loaded into Fiji and applied to images of costained DRGs. Somatic fluorescence was evaluated using the standard ROIs. To detect DBI, the ROIs were overlaid onto the DBI fluorescence channel using the ImageJ plug-in “Labels to ROIs.” This plug-in allowed us to change the size of ROIs by enlarging or shrinking them. Each original ROI was dilated by 10 pixels added radially to obtain the fluorescence of the whole cell plus an area slightly outside the cell. Successively, each original ROI was shrunk by removal of 3 pixels radially for maximal capture of pericellular DBI fluorescence. To calculate the specific glial DBI pericellular fluorescence, the eroded ROIs were subtracted from the enlarged ones, producing a doughnut-shaped area used for quantification of the DBI fluorescence. Raw integrated density values were normalized by the area selected, and the data are presented as “mean fluorescence.”

### Live fluorescence iodide imaging

HEK293 cells were cotransfected with cDNA encoding human α_1_, β_2_, and γ_2_ GABA_A_ receptor subunits and the halide-sensitive EYFP mutant (H148Q/I152L; EYFP-QL). Transfected cells were cultured alone or cocultured with the purified SGCs for 24 hours. Extracellular solution consisted of (in mM): 160 NaCl, 2.5 KCl, 1 MgCl_2_, 2 CaCl_2_, 10 HEPES, and 10 glucose (pH adjusted to 7.4 with NaOH; all from Sigma-Aldrich). I^–^-containing solution was produced by equimolar substitution of 5 mM NaCl with NaI. I^–^ imaging was performed using a Nikon Eclipse TE-2000-S microscope and an imaging system consisting of a Polychrome V monochromator and an IMAGO CCD camera controlled with Live Acquisition 2.2.0 software (FEI). Cells were located by bright-field and epifluorescence (excitation at 488 nm). Data were collected and analyzed using Offline Analysis (2.2.0) software (FEI).

### Patch clamp recording

Whole-cell patch clamp recordings from mouse and rat primary DRG neurons and transfected HEK293 cells were performed with a MultiClamp 700B amplifier/Digidata 1440 and pCLAMP 10.0 software (Molecular Devices) or using EPC10 amplifier and Patchmaster/Fitmaster v2 software (HEKA Instruments) at room temperature. Cells were continuously perfused at approximately 2 mL/min with the extracellular bath solution containing (in mM): 160 NaCl, 2.5 KCl, 5 CaCl_2_, 1 MgCl_2_, 10 HEPES, and 8 glucose (pH 7.4); the intracellular solution contained (in mM): 150 KCl, 5 MgCl_2_, 10 HEPES (pH adjusted to 7.4 with KOH). Osmolarity was adjusted to 290–300 mOsm. For the perforated patch recording the pipette solution contained (in mM): 120 K-acetate, 30 KCl, 5 MgCl_2_, 10 HEPES, including 300 μg/mL of amphotericin B, daily made (pH adjusted to 7.4 with KOH; all from Sigma-Aldrich). The recording electrodes (resistance 3–5 MΩ) were fabricated from borosilicate glass capillaries using a DMZ-Universal horizontal puller (Zeitz) or a P-97 puller (Sutter) and fire-polished. Capacitance artifacts were canceled and series resistance compensated by 60%–70%. Continuous gap-free voltage-clamp recordings were performed at a holding potential of –60 mV. Current-clamp mode was used to record action potential waveforms from DRG neurons following injection of 500-millisecond depolarizing steps (30 pA).

### Focal application of substances to DRG in vivo

#### DRG cannula.

A DRG cannula for focal application of substances to DRG was implanted as previously described (2). Briefly, a midline incision was made at the L3 –L5 spinal level of adult male mice (C57BL/6; 20–25 g), and the L4 was identified at the midpoint of a link between both sides of the iliac crest. A 0.8 mm hole (about 1 mm off the inferior edge of the transverse process) was drilled through the transverse process over the L4 DRG. Approach of a ganglion was verified by a twitch of the paw. A hooked stainless steel blunt-tip cannula (inner diameter, 0.64 mm; length, 4 mm) was forced into the hole and connected to a polypropylene tube (inner diameter, 0.41 mm; length, 4.5 mm). The incision was closed with sutures, and the cannula was firmly fixed in place with dental cement. Intramuscular injection of benzylpenicillin (19 mg/0.1 mL) was given immediately after surgery. Postoperatively, mice were housed individually in plastic cages with sawdust flooring and supplied with water and food ad libitum. Animals were left to recover for at least 24 hours before the experiments were performed.

#### DRG osmotic minipumps.

Osmotic minipumps (ALZET model 2002) were implanted in a procedure similar to the DRG cannula implant. The pumps were implanted s.c. in the neck while the cannula tube (ALZET Brain Infusion Kit 2) connected to the pump was inserted into the vertebral transverse process over the L4 DRG. Before the implantation, the infusion assembly with attached osmotic pump was incubated in sterile saline (0.9%) at 37°C for 6 hours. This pump model releases approximately 0.5 μL/h for 14 days. All surgical procedures were performed under deep anesthesia with an intraperitoneal injection of sodium pentobarbital (40–60 mg/kg) in accordance with the Animal Care and Ethical Committee of Hebei Medical University under the International Association for the Study of Pain guidelines.

#### Viral injections.

AAV9-U6-shDBI-CAG-EGFP, AAV5-gfaABC1D-DBI-EGFP, and AAV5/9-null-EGFP virions were obtained from Genechem Co. Ltd. (Shanghai, China). AAV virions were injected into the right-side L4 DRG of C57BL/6 male mice as previously described (2). Briefly, under deep anesthesia (sodium pentobarbital, 40–60 mg/kg, i.p.), L4 DRGs of adult male mice were exposed by removal of both spinous and transverse processes of the vertebra bone. The microinjector (Hamilton Co.) was inserted into the ganglion to a depth of approximately 300 μm from the exposed surface. The virion solution (1.1 × 10^12^ to 1.2 × 10^12^ viral genomes/mL; 2 μL) was injected slowly, and the needle was removed 5 minutes after the injection was complete. The muscles overlying the spinal cord were loosely sutured together, and the wound was closed. Thermal and mechanical sensitivity tests were performed before and at scheduled intervals after the injection. After the behavioral testing, mice were humanely sacrificed and DRGs extracted for EGFP visualization.

### Intrathecal delivery of substances.

Intrathecal injections were performed by a spinal puncture made with a 30-gauge needle between the L5 and L6 levels, as described previously (71, 72). Mice received an intrathecal injection of siRNAs targeting *Dbi* (s64829), *Gabra1* (s201367), and *Gabrg2* (s66392) at 2 μg of siRNA per delivery in the transfection agent polyethylenimine; a non-targeting control siRNA (4390846) was used as negative control (all siRNA oligonucleotides were from Thermo Fisher Scientific). Recombinant human DBI protein (Abcam, ab84342) was intrathecally injected at 10 ng per delivery. Behavioral tests were performed 48 hours after the injection.

### In vivo recording of peripheral nerve and dorsal root activity

These recordings were performed as described previously (3). All surgical procedures were performed under deep anesthesia with an i.p. injection of sodium pentobarbital (40–60 mg/kg). Laminectomy was performed to expose right L4 DRG of adult male C57BL/6J mice. The dorsal root, the DRG, and the spinal nerve outlet were exposed by removal of both spinous and transverse processes of the vertebra bone; the nerve outlets were then suspended on the hooked stainless steel recording electrodes connected to a BL-420F biological data acquisition and analysis system (Chengdu Techman Software Co.). The wound was filled with paraffin. The right hind paw was stimulated with hot water (approximately 60°C), von Frey filament (0.6 g), or light prick with a syringe needle, avoiding piercing the skin. DBI (200 μM; 2 μL) was accurately delivered to the surface of exposed DRG by micropipettor.

### Chronic pain models

Complete Freund’s adjuvant (CFA) inflammatory pain model and spared nerve injury (SNI) (73) neuropathic pain model were used. Details are provided in Supplemental Methods.

### Behavioral tests

Mechanical sensitivity was tested using the von Frey up-down method (74). Adhesive removal assay was performed as described previously (75, 76). Other tests included pinprick response test, alligator clip test (76), cold allodynia (dry ice test; ref. 77), Hargreaves assay for heat sensitivity (78), and rotarod test. See Supplemental Methods for details.

### Statistics

Summary data are presented as mean ± SEM with statistical significance assessed by 2-tailed Student’s *t* test, Mann-Whitney test, or 1- or 2-way ANOVA with Tukey’s multiple-comparison test where appropriate. Comparison of 3 or more groups in behavioral data was analyzed using repeated-measures ANOVA with Šidák’s post hoc test. *P* ≤ 0.05 was adopted as an indicator of statistically significant difference between means. Statistical analysis was performed using GraphPad Prism 9.0 software.

### Study approval

#### Animals.

Animal experiments were performed using adult male and female CD1 (Charles River) and C57BL/6J (Beijing Vital River Laboratory Animal Technology Co. Ltd.) mice aged 6–12 weeks or male and female Wistar rats (150–250 g; University of Leeds animal facility). Animals were given ad libitum access to food and water and housed in a controlled environment following the institutional guidelines and the Animal Research: Reporting of *In Vivo* Experiments (ARRIVE) guidelines (79). Animal experiments performed at Hebei Medical University were in accordance with the Animal Care and Ethical Committee of Hebei Medical University (Shijiazhuang, China; approval IACUC-Hebmu-2020007). Animal work carried out at the University of Leeds was approved by the University of Leeds Animal Welfare and Ethical Review Committee and performed under the UK Home Office License P40AD29D7 in accordance with the regulations of the UK Animals (Scientific Procedures) Act 1986. Animal experiments conducted at the University of Cincinnati were approved by the Institutional Animal Care and Use Committee of the University of Cincinnati (22-01-03-02) and conducted in accord with the NIH’s *Guide for the Care and Use of Laboratory Animals* (National Academies Press, 2011). The sample size was determined based on our previous studies. Whenever possible, animals were randomly assigned to different experimental groups, and the investigators were blinded to animal treatment allocations.

#### Human DRGs.

Adult human DRGs were collected from healthy deidentified organ donors with the approval of the University of Cincinnati Institutional Review Board (IRB 021-0287). Fetal human DRGs were obtained from a donor who gave written informed consent at the Third Hospital of Hebei Medical University and collected in *N*-methyl-d-glucamine artificial cerebrospinal fluid (NMDG-aCSF) following thoracolumbar spine harvest (T10/11 to L5/S1). Collection of human DRGs was approved by the Clinical Research Ethics Committee of Hebei Medical University (IRB 20190040).

### Data availability

Most data are included in the figures as scatterplots; all metadata are available in the Supporting Data Values file; source data are available upon request.

## Author contributions

XL, ASP, and VP performed experiments, analyzed data, and drafted the manuscript. XG performed experiments. HH, FJ, SF, and PM performed experiments and analyzed data. YW, RT, SHL, SS, and BM performed experiments. TB, XD, and NG designed the study, analyzed data, and wrote the manuscript. The order of co–first authors was chosen based on the relative volume of data contributed.

## Supplementary Material

Supplemental data

Unedited blot and gel images

Supplemental video 1

Supporting data values

## Version 1 06/18/2024

In-Press Preview

## Version 2 08/15/2024

Electronic publication
