# Supplementary material for: Peripheral gating of mechanosensation by glial diazepam binding inhibitor
Source: J Clin Invest. 2024 Jun 18;134(16):e176227. doi: 10.1172/JCI176227 (PMC11324294; doi:10.1172/JCI176227)
Supplement: Supplemental data [file jci-134-176227-s119.pdf]

Supplemental Material

Supplemental Methods

Chronic pain models.

Complete Freund's Adjuvant (CFA). To induce chronic inflammatory pain, mice were injected with 20 μl of complete Freund's adjuvant (Sigma) in the glabrous surface of the right hind paw, and sterile 0.9% NaCl was injected into the control mice.

Spared nerve injury (SNI). SNI was performed following the procedure described previously (73). Briefly, mice were deeply anesthetized by sodium pentobarbital (40–60 mg/kg, i.p). Skin on the lateral surface of the right thigh was incised and underlying muscle was opened by blunt dissection to expose the three branches of the sciatic nerve. The peroneal and tibial branches were tightly ligated with 5–0 silk and transected below the ligature, and a 2–3 mm section distal to the ligature was removed, leaving the sural nerve intact. The muscle tissue and the skin were closed with sutures.

Behavioural tests

Mechanical threshold. Mice were acclimated for 30-60 min in von Frey testing chambers. Fifty percent mechanical threshold was measured with calibrated von Frey filaments (0.02g, 0.04g, 0.07g, 0.16g, 0.4g, 0.6g, 1.0g, 1.4g, 2.0g and 4.0g), which were applied directly under the glabrous skin of the hindpaw until the hairs bent slightly. Clear withdrawal within 1 second of applying the hair was considered as a response. Each filament was applied four times using the up-down method (74).

Adhesive removal assay (Tape assay). The latency to bite, lick, or attempt removal of a circular adhesive was measured as previously described (75, 76); the stimulus was applied to the hind paw only. Briefly, mice were first acclimated in a round plexiglass container for at least 5 minutes. A 9-mm diameter circular adhesive Microtube Tough-Spots label (Diversified Biotech) was delicately affixed on the plantar surface of the hind paw. The mice were observed for 5 minutes, and the total

time spent responding to the tape was recorded. Biting, scratching, or visibly attempting to remove the tape from the paw was scored as a response.

Response to pinprick. Mice were acclimated in von Frey testing chambers for 1 hour. A 27-gauge needle was applied to the glabrous skin of the hindpaw, taking care not to pierce through the skin. Ten trials per mouse were performed with 1-min intervals between each trial. Paw withdrawal, shaking, or licking was scored as a response and reported as percentage for the total number of trials. A new needle was used for each mouse.

Alligator clip. Response to blunt pressure application was assessed with the alligator clip assay, as previously described (76), modified to hind paw. Briefly, mice were first acclimated for 5 min in round plexiglass containers. An alligator clip (with constant pressure) was applied to the middle of the paw, and the mouse was placed back into the plexiglass container. A response was scored when the mouse showed awareness of the clip by biting, vocalization, grasping of the paw, or a jumping response. A cut-off of 90 s was applied to prevent tissue damage. The time to respond was recorded and reported as latency for each mouse.

Cold allodynia (dry ice test). The test was performed as previously described (77). Each animal was placed in a clear acrylic container separated by black opaque dividers, which were positioned on top of 3/16" borosilicate glass (Stemmerich Inc, USA) and allowed to acclimate for 20 minutes before testing. A dry ice pellet was applied to the hind paw through the glass and the time until hind paw withdrawal was recorded; three trials were performed for each mouse at 5-min intervals and mean withdrawal latency was calculated.

Hargreaves assay. Heat sensitivity was assessed using the Hargreaves method (78). The radiant heat source was applied to the plantar surface of the mouse hind paw and the latency to withdrawal was used to determine the heat sensitivity threshold, with a cut-off of 20 s to prevent tissue damage; three trials were performed for each mouse at 5-min intervals and mean withdrawal latency was recorded.

Rotarod assay. Motor function was assessed using a rotarod apparatus (IITC Life Science) and measured as the latency of the mouse to fall off the rotating platform, with an acceleration of 0.2 rpm/sec over a two-minute period.

Quantitative real-time RT-PCR (qPCR). DRGs were extracted and total RNA was extracted using Trizol reagent (Invitrogen). Isolated RNA was dissolved in 10 μl of DEPC-treated water and reverse-transcribed using a reverse transcription reagent kit (PrimeScript RT Reagent Kit with gDNA Eraser, Takara) and a thermal cycler (Mastercycler, Eppendorf). Quantitative PCR (qPCR) analysis was performed in the Real-time Detection System (FQD-48A(A4), BIOER) by SYBR Premix Ex TaqII (Takara). The PCR products were also run on a 2% agarose gel and were visualized using a gel imager (TFP-M/WL, Vilber Lourmat). Primers for qPCR analysis are listed in Suppl. Table 1.

Enzyme Linked Immunosorbent Assay (ELISA). DRGs from all spinal levels of C57BL/6 mice were rapidly extracted into HBSS on ice and washed once. Standard DRG incubation solution (500 μl) containing (in mM):160 NaCl, 2.5KCl, 5$CaCl_2$, 1 $MgCl_2$, 10 HEPES, 8 D-glucose (pH adjusted to 7.4 with NaOH; all from Sigma) was added and the ganglia were incubated for 30 minutes at room temperature. The 'High-K^+' solution was produced by equimolar replacement of 150 mM NaCl with 150 mM KCl. After incubation, the supernatants were collected for DBI detection using DBI ELISA kit from Abbexa Ltd (abx153899), according to the manufacturer's instructions. Fluostar Omega microplate reader (BMG LABTECH, Germany) was used to analyse the samples.

Supplemental Table 1. Antibodies, probes and DNA constructs

REAGENT	SOURCE	Dilution	IDENTIFIER
Antibodies			
Rabbit anti-DBI	Abcam	1:1000	ab231910
Rabbit anti-DBI	Frontier institute	1:400	AB_2571690
Rabbit anti-FABP7	Thermo Fisher Scientific	1:400	PA524949
Mouse anti-FABP7	Neuromics	1:500	MO22188
Goat anti-IBA-1	Novus Biologicals	1:1000	NB1001028
Rabbit anti-Glutamine Synthetase	Abcam	1:1000	ab49873
Rabbit anti-NeuN	Abcam	1:1000	ab177487
Rabbit anti-NF200	MilliporeSigma	1:250	N4142
Rabbit anti-Peripherin	Novus Biologicals	1:1000	NB300-137
Mouse anti-beta III Tubulin	Abcam	1:1000	ab78078
Mouse anti-beta III Tubulin	BioLegend	1:400	801201
Mouse anti-S100B	Novus Biologicals	1:100	NBP2-29403
α1 $GABA_A$	Invitrogen	1:500	PA5-114315
γ2 $GABA_A$	Synaptic-systems	1:200	224004
Mouse anti-CD31	R&D Systems	1:500	AF3628
Anti-mouse Alexa Fluor 488	Thermo Fisher Scientific	1:600	A21202
Anti-goat Alexa Fluor 555	Thermo Fisher Scientific	1:1000	A21432
Anti-goat Alexa Fluor 594	Thermo Fisher Scientific	1:600	A11058
Anti-mouse Alexa Fluor 594	Thermo Fisher Scientific	1:1000	A11005
Anti-rabbit Alex Fluor 546	Thermo Fisher Scientific	1:1000	A11010
RNAscope probes			
Dbi	Advanced Cell Diagnostics		502601
Dbi	Advanced Cell Diagnostics		502601-C2
Gabra1	Advanced Cell Diagnostics		435351-C3
Gabrg2	Advanced Cell Diagnostics		408051-C2
Piezo2	Advanced Cell Diagnostics		400191
Trpv1	Advanced Cell Diagnostics		313331
Trpv1	Advanced Cell Diagnostics		313331-C3
Oligonucleotides			
5'*GAPDH* F:CTCAGGAGAGTGTTTCCTCG	Sangon Biotech		N/A
5'*GAPDH* R:TTTGCCGTGAGTGGAGTCAT	Sangon Biotech		N/A
5'*DBI* F:GTGCGCTCTGTGACTTGATT	Sangon Biotech		N/A
5'*DBI* R:CCCCGGCCGATCTGTATTTA	Sangon Biotech		N/A
cDNA constructs			
Human *GABRA1, GABRAG2, GABRB2*	gift of David Weiss, Department of Physiology, University of Texas Health Science Center, San Antonio, Texas, USA		N/A
Mouse $GABA_A$ receptor subunits and mutants	Sangon Biotech		N/A

Supplemental Figures

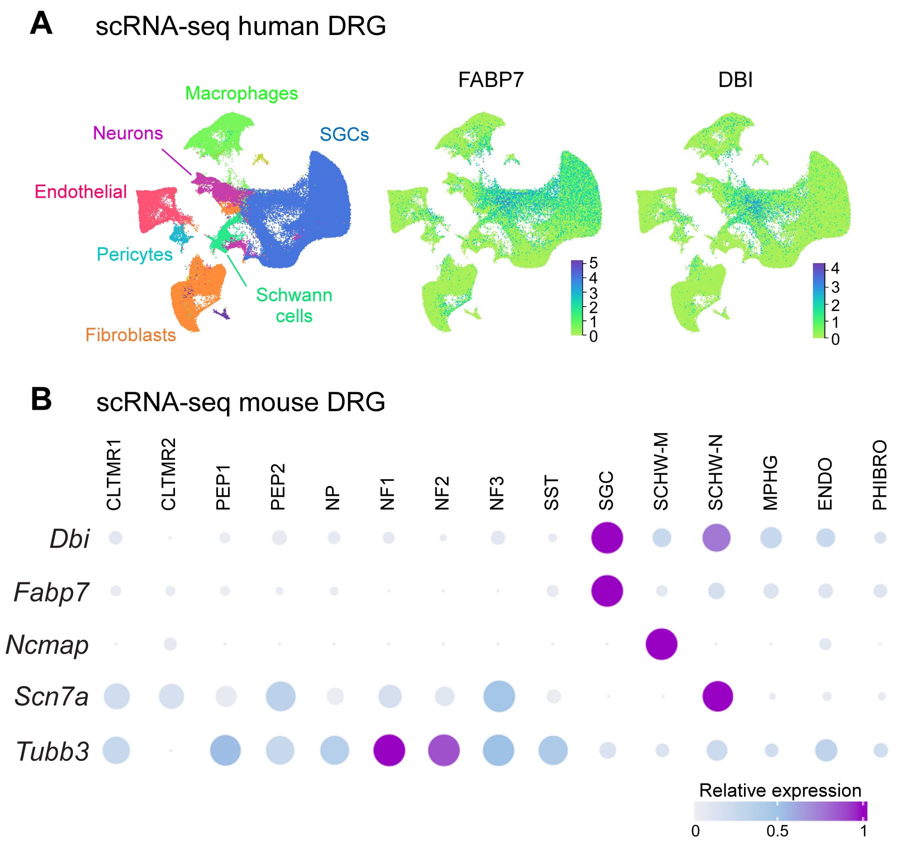

Supplemental Figure 1. Analysis of DBI expression profile from existing transcriptomic databases. A, Analysis of DBI expression in the human nervous system based on single-cell transcriptomic data from (37). DBI co-clustered with the SGC marker, FABP7 and displayed less abundant distribution with other DRG-resident cell types, including neurons. B, Analysis of DBI expression in the mouse DRG based on single-cell transcriptomic data from (39). DBI is strongly expressed in SGC, moderately expressed in non-myelinating Schwann cells; expression in other DRG-resident cell types is low. Also shown the expression patterns of cell markers for SGC (*Fabp7*), myelinating Schwann cells (*Ncmap*), non-myelinating Schwann cells (*Scn7a*) and a pan-neuronal marker (*Tubb3*).

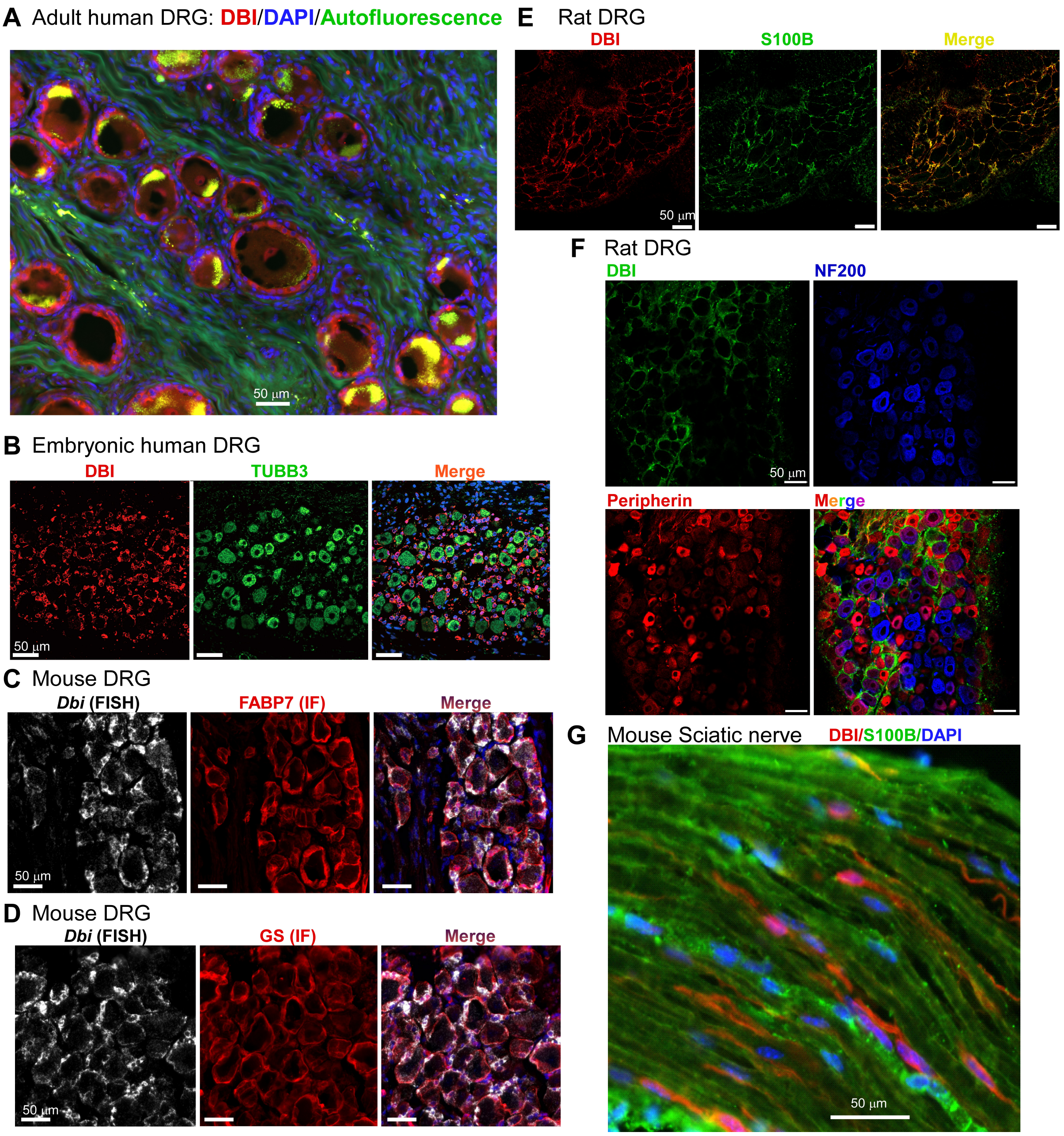

Supplemental Figure 2. Additional data identifying DBI expression pattern in DRG. A, Immunofluorescence (IF) staining of a section from an adult human DRG (red – DBI, blue – DAPI, green – autofluorescence). **B**, IF staining of a section from a foetal human DRG (red – DBI, green – TUBB3, blue – DAPI). **C**, **D**, Combined fluorescence in situ hybridisation (FISH) and IF analysis of mouse DRG sections. Co-labelling of DBI (FISH, white) and FABP7 (IF, red) is shown in (C); Co-labelling of DBI (FISH, white) and GS (IF, red) is shown in (D). **E**, IF co-labelling rat DRG sections with antibodies against DBI (red) and S100B (green). **F**, IF co-labelling of rat DRG neuron section with antibodies against DBI (green), NF200 (blue) and peripherin (red). **G.** IF co-labelling of mouse sciatic nerve section with antibodies against DBI (red) and S100B (green).

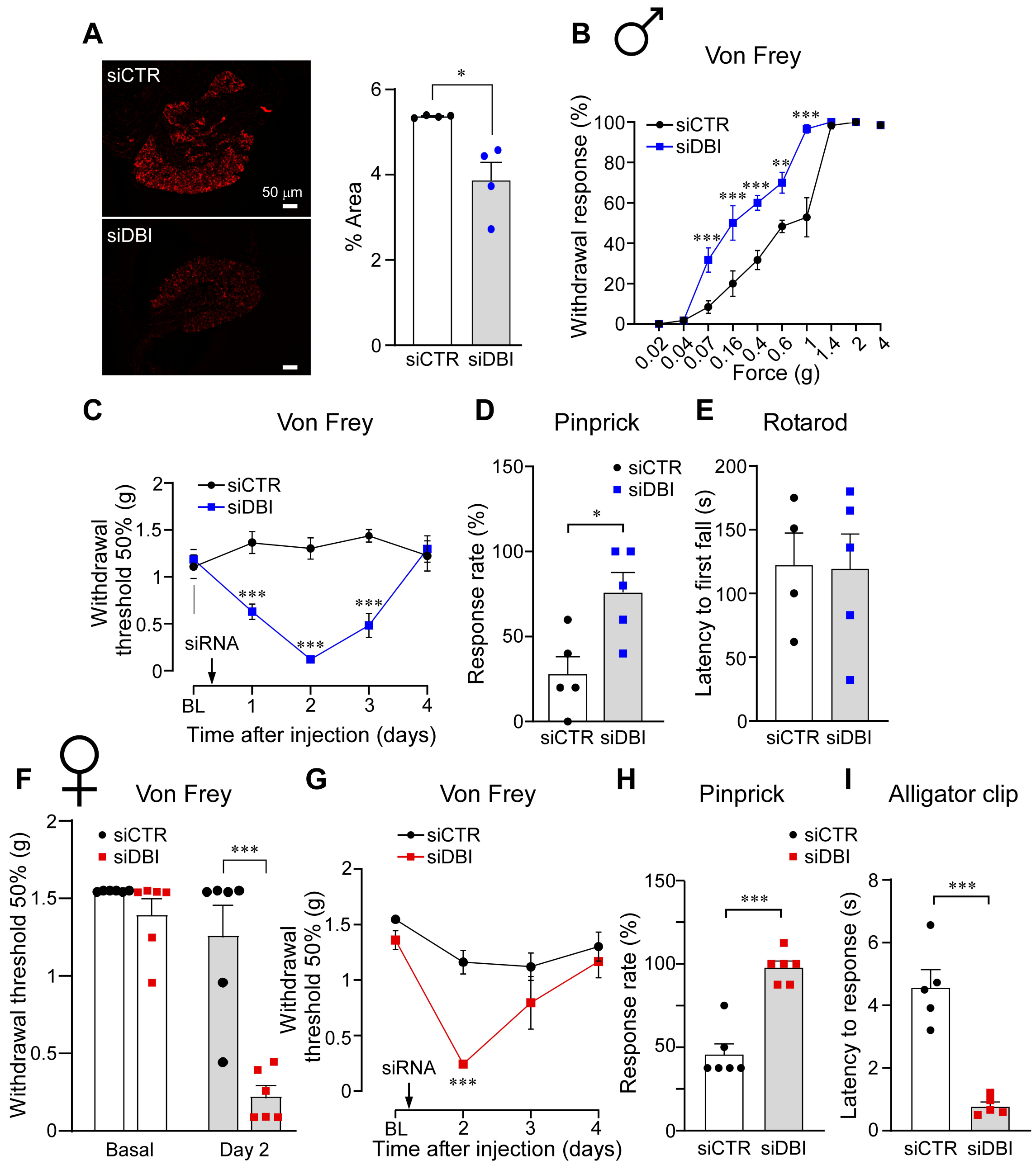

Supplemental Figure 3. Additional DBI siRNA knockdown experiments. A, Confirmation of the intrathecal siRNA knockdown efficiency of DBI; example immunofluorescence staining of DRG sections from mice receiving siRNA against *Dbi* or non-targeting control oligonucleotide (2 µg/site). Immunofluorescence was quantified as percentage of immunolabeled area per section. **B-E**, Experiments on male mice. **B**, Analysis of the von Frey force vs. withdrawal response rate for mice intrathecally injected with either siRNA against *Dbi* (2 µg/site; blue) or non-targeting control oligonucleotide (black); behavioural tests were performed 48 h after the injection. **C**, Timeline of the mechanical sensitivity changes following a single intrathecal injection of siRNA against *Dbi* (blue) or non-targeting control oligonucleotide (black). In (A, B) **, *** indicate significant difference from matched control group ($p<0.01$, $p<0.001$; two-way repeated-measures ANOVA with Sidák post-hoc test). **D**, **E**, Summary of the results of the pinprick (C) and rotarod test (D) of mice intrathecally

injected with either siRNA against *Dbi* (blue symbols) or non-targeting control oligonucleotide (black symbols); behavioural tests were performed 48 h after the injection; *indicates significant difference between the siDBI and control groups ($p<0.05$; unpaired t-test). **F-I**, Experiments on female mice. **F**, Summary of the von Frey tests before and 48 h after the intrathecal injection of either siDBI (red symbols) or control oligonucleotide (black symbols); ***indicates significant difference between the siDBI and control groups ($p<0.05$; unpaired t-test). **G**, Experiment similar to that in panel (B) but conducted on female mice. ***indicates significant difference from matched control group ($p<0.001$; two-way repeated-measures ANOVA with Sidák post-hoc test). **H**, **I**, Summary of the results of the pinprick (G) and the alligator clip test (H) of female mice intrathecally injected with either siDBI (red symbols) or control oligonucleotide (black symbols); behavioural tests were performed 48 h after the injection; ***indicates significant difference between the siDBI and control groups ($p<0.001$; unpaired t-test).

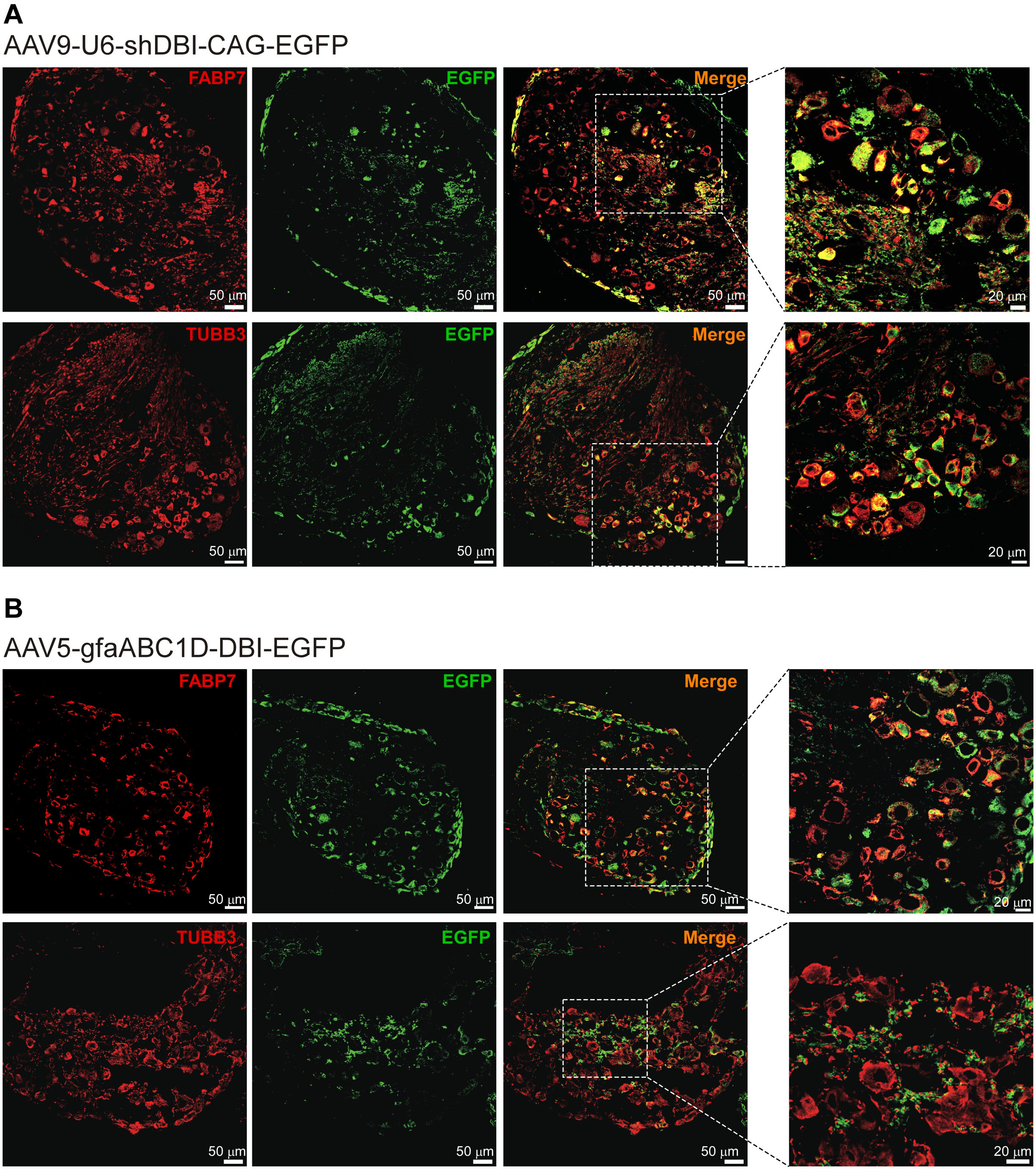

Supplemental Figure 4. Analysis of transgene expression in the DRG following viral construct injections. A, Examples of fluorescence micrographs of mouse DRG 8 weeks after DRG injection with AAV9-U6-shDBI-CAG-EGFP virions; GFP fluorescence (green) and FABP7 (upper panels) and TUBB3 (lower panels) immunofluorescence (red) are shown individually (as labelled) or overlaid ('merge'). **B**, Examples of fluorescence micrographs of mouse DRG 8 weeks after DRG injection with AAV5-gfaABC1D-DBI-EGFP virions. Presentation and labelling as in panel A.

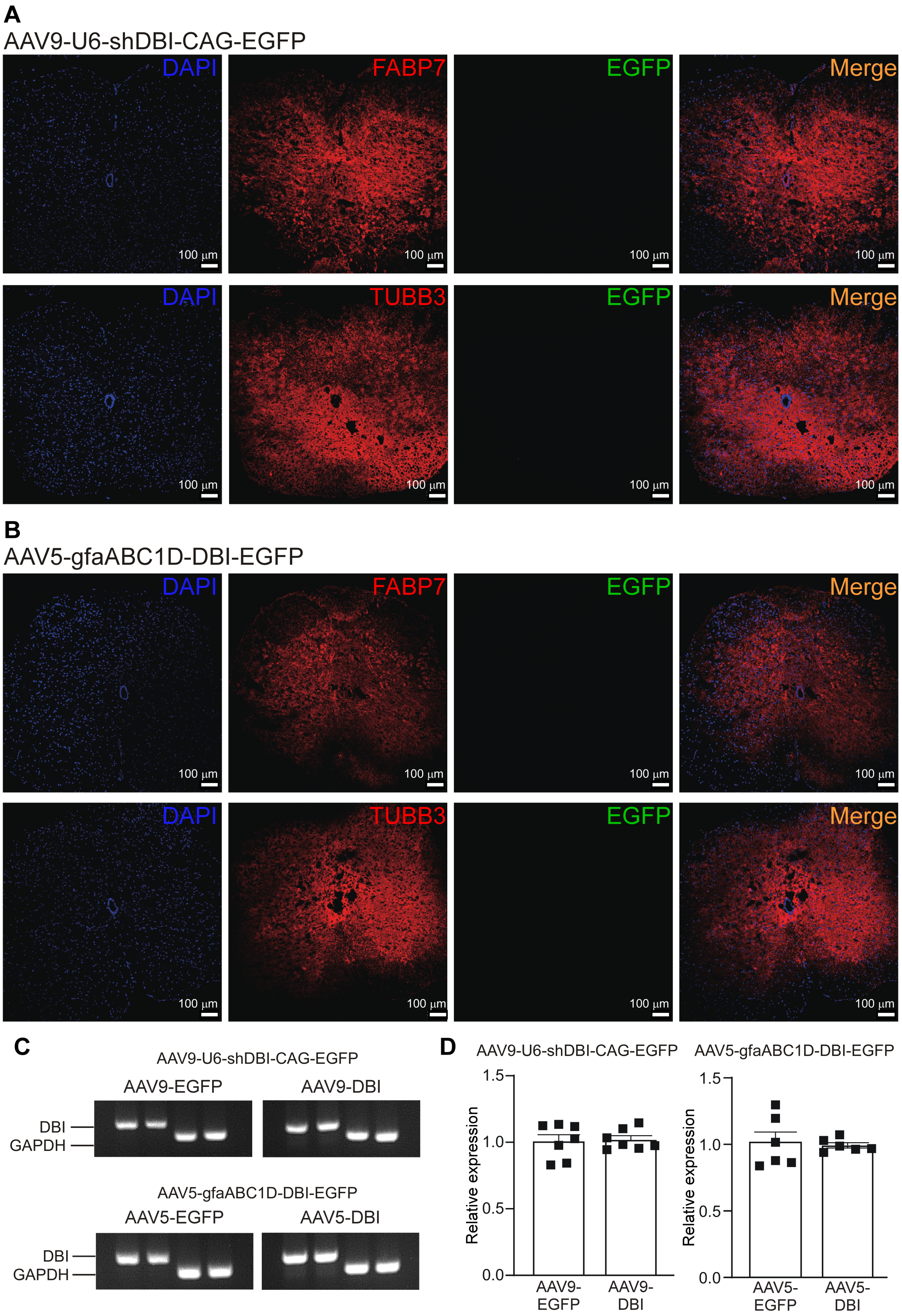
A
AAV9-U6-shDBI-CAG-EGFP
DAPI
FABP7
EGFP
Merge
TUBB3
100 μm
B
AAV5-gfaABC1D-DBI-EGFP
C
AAV9-EGFP
AAV9-DBI
DBI
GAPDH
AAV5-EGFP
AAV5-DBI
D
Relative expression
1.5
1.0
0.5
0.0
AAV9-EGFP
AAV9-DBI
AAV5-EGFP
AAV5-DBI

Supplemental Figure 5. Genetic manipulations of DBI expression by viral DRG injections do not significantly affect spinal levels of DBI. A, B, GFP fluorescence (green) is not detectable in the spinal cord sections corresponding to L4 DRG of mice 8 week after DRG injection with either AAV9-U6-shDBI-CAG-EGFP virions (A) or AAV5-gfaABC1D-DBI-EGFP virions (B). Co-immunolabelling with FABP7 (upper panels) and TUBB3 (lower panels) is shown in red; DAPI nuclear staining is shown in blue. **C**, **D**, Example RT-PCR analyses (C) and summarised RT-PCR data (D) on the *Dbi* transcript level in the lumbar spinal cord sections of mice 8 week after DRG injection with either AAV9-U6-shDBI-CAG-EGFP virions, or AAV5-gfaABC1D-DBI-EGFP virions (as indicated).

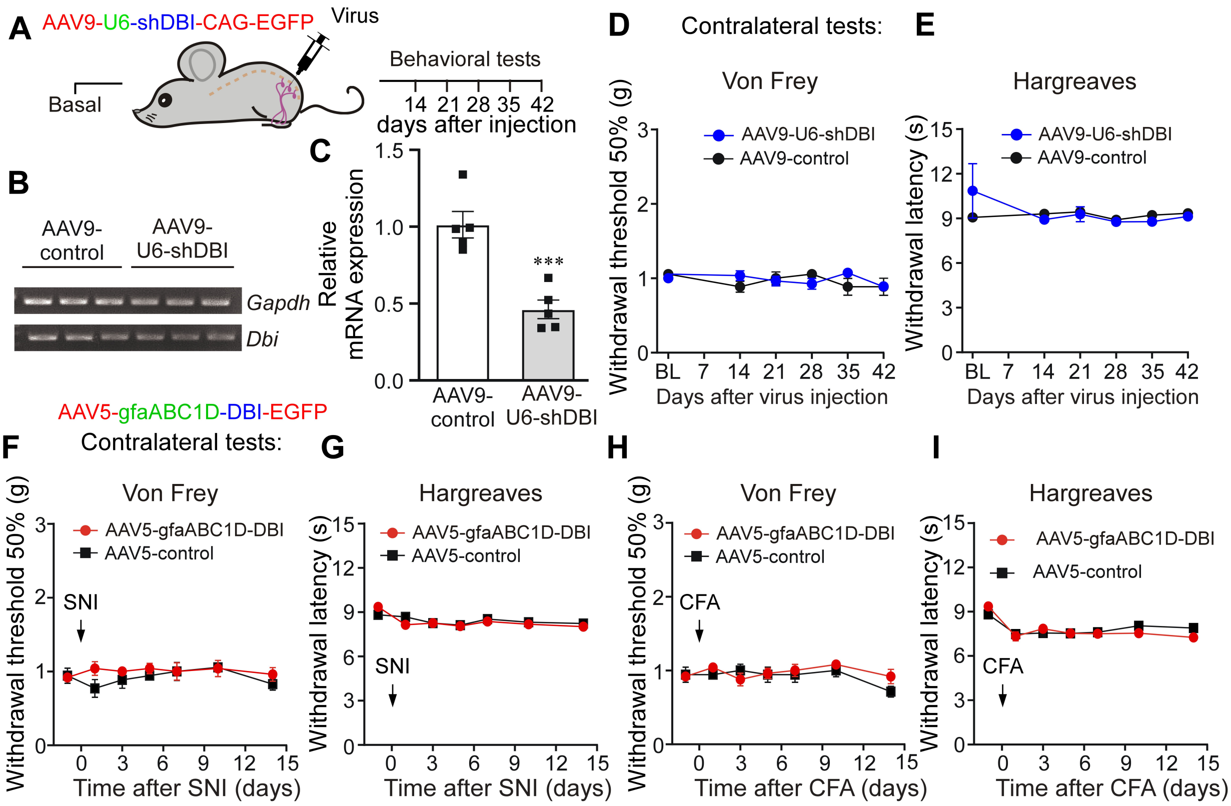

Supplemental Figure 6. Additional data for the manipulations with DBI expression by DRG-delivered viral constructs. A, Schematic of the experimental timeline for the experiments with DRG injection AAV9-U6-shDBI-CAG-EGFP virions (1.1 - 1.2x10^{12} vg/ml; 2 µl). **B**, **C**, Example RT-PCR analyses (B) and summarised RT-PCR data (C) on the *Dbi* transcript levels in the DRG after control or AAV9-U6-shDBI-CAG-EGFP virion injection; ***indicates significant difference from control group ($p<0.001$, unpaired t-test). **D**, **E**, Mechanical (von Frey) and thermal (Hargreaves) sensitivity tests during 42 days after the DRG injection of AAV9-U6-shDBI-CAG-EGFP virions or GFP control virions performed on the contralateral paws; the results of the corresponding ipsilateral paw tests are shown in Fig. 2H, I. **F-I**, Contralateral paw mechanical (von Frey) and thermal (Hargreaves) sensitivity tests on mice received DRG injections of AAV5-gfaABC1D-DBI-EGFP virions or GFP control virions (1.1 - 1.2x10^{12} vg/ml; 2 µl). Six weeks after viral injections mice underwent either SNI surgery (F, G) or hind paw CFA injections (H, I). The mice were tested for mechanical and thermal sensitivity thresholds during subsequent 14 days; the results of the corresponding ipsilateral paw tests are shown in Fig. 3G-J.

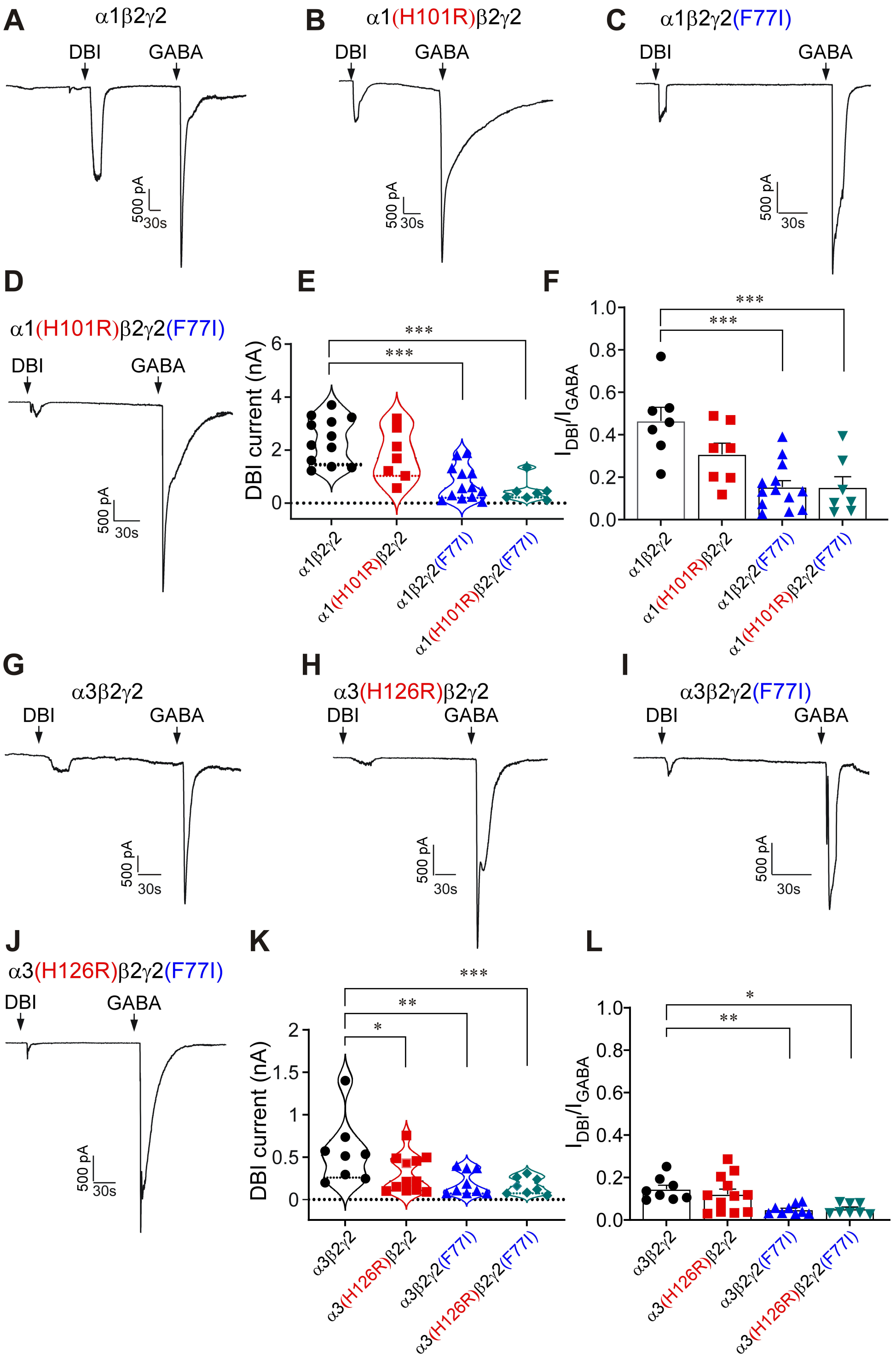

Supplemental Figure 7. Additional experiments testing DBI agonism at GABA$_A$ receptors. A-D, Example current traces of whole-cell patch-clamp recordings from HEK293 cells heterologously transfected with mouse α1, β2 and γ2 GABA$_A$ subunits, as well as with α1(H101R) or γ2(F77I) or their combination (as indicated). Recombinant purified DBI (200 nM) or GABA (200 µM) were applied via localised perfusion system at times depicted by the black arrows. **E**, Summarised amplitudes of DBI-induced inward currents for experiments exemplified in panel (A). **F**, Summarised ratios of DBI-induced to GABA-induced current amplitudes. In (E, F) ***indicates significant difference from α1β2γ2 GABA$_A$ group ($p<0.001$, one-way ANOVA with Dunnett post-hoc test). **G-**

J, Similar to panels (A-D) but α3β2γ2 $GABA_A$ channels were studied, together with α3(H126R) or γ2(F77I) or their combination (as indicated); other conditions and labelling as in panels (A-D). **K**, **L**, summarise the data from experiments in (G-J), similar to panels (E, F). *,**,***indicate significant difference from α3β2γ2 $GABA_A$ group ($p<0.05$, $p<0.01$, $p<0.001$, respectively; one-way ANOVA with Dunnett post-hoc test).

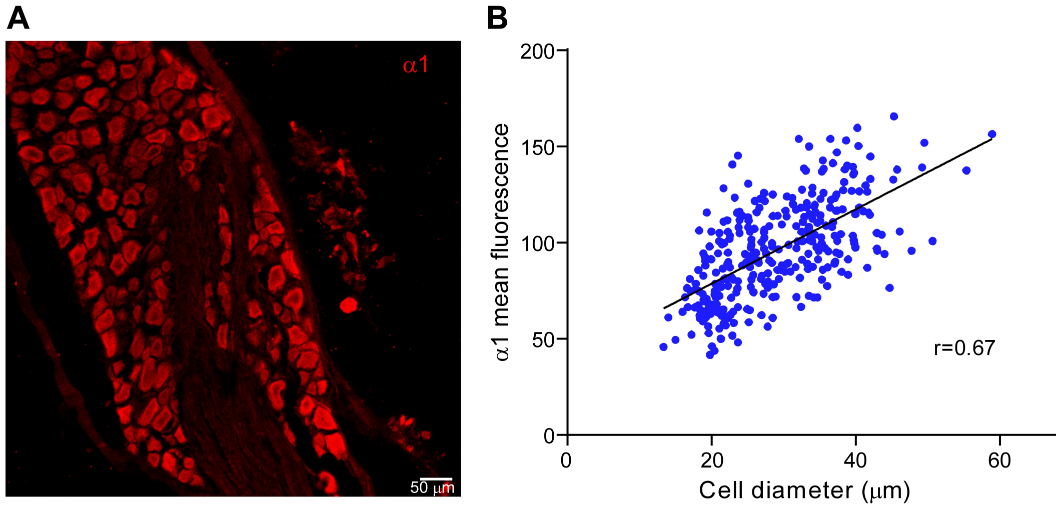

Supplemental Figure 8. Correlation between the somatic diameter of the DRG neurons and α1 GABA$_A$ subunit immunoreactivity. A, Example immunofluorescence staining of the rat DRG section with the antibody against α1 GABA$_A$ subunit (red). **B**, Correlation between the somatic diameter of the DRG neurons and α1 IF integrated density.

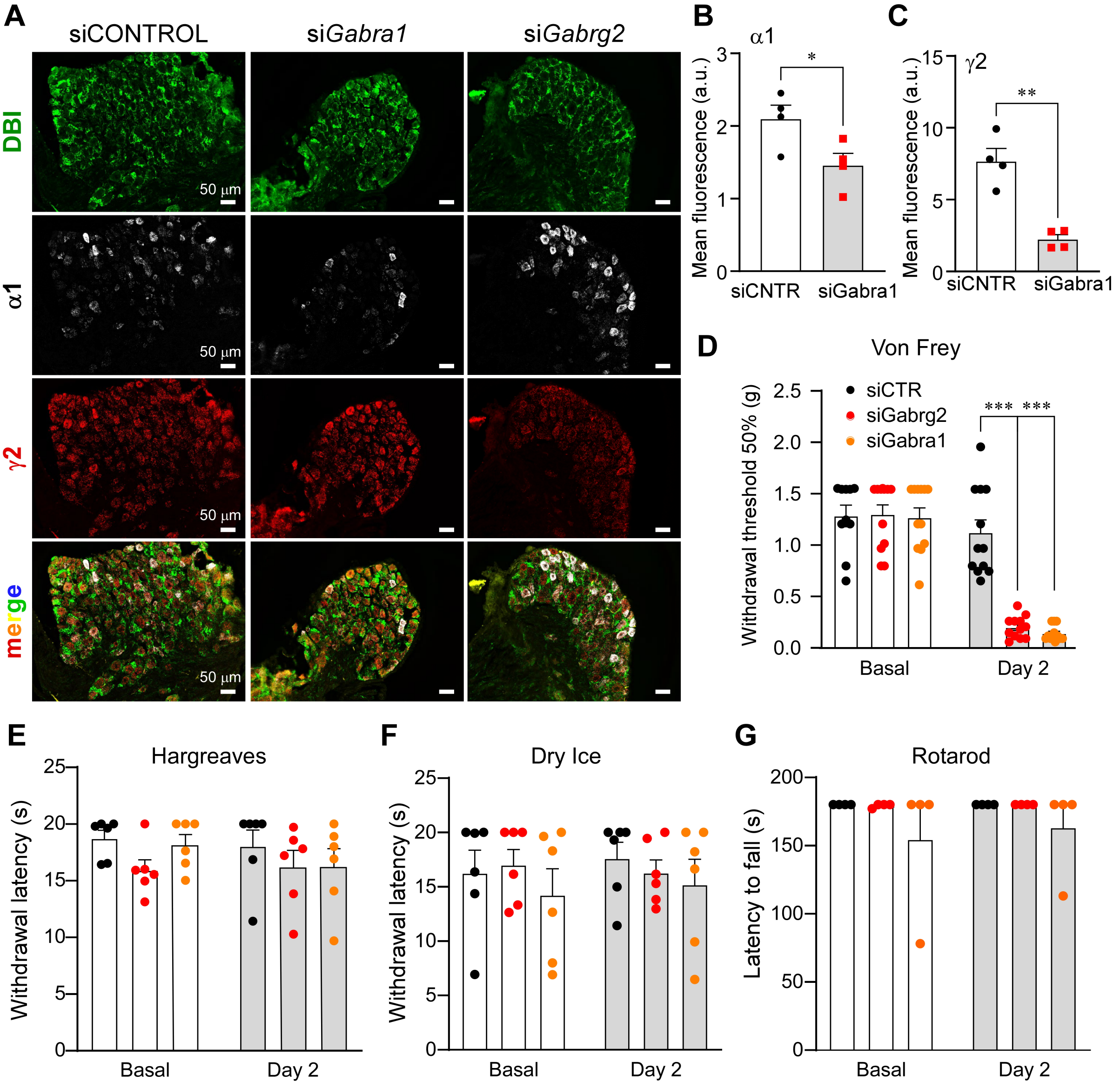

Supplemental Figure 9. Knockdown of α1 and/or γ2 GABA$_A$ subunits in DRG recapitulates mechanical hypersensitivity produced by the DBI knockdown. A-C, Confirmation of the intrathecal siRNA knockdown efficiency for siRNAs targeting *Gabra1* and *Gabrg2*. **A**, example immunofluorescence staining of DRG sections from mice receiving siRNA against *Gabra1*, *Gabrg2* or non-targeting control oligonucleotide (2 μg/site), (DBI – green, α1 – white, γ2 – red); staining was performed 48 h after intrathecal injections. In (B) and (C) immunofluorescence was quantified as the fluorescence intensity normalised to each section's area. **D-G**, siRNA against *Gabra1* (yellow symbols), *Gabg2* (red symbols) or a non-targeting control siRNA (black symbols) were intrathecally injected (2μg/site) and 48h later the following tests were performed: mechanical sensitivity (von Frey) test (D), Hargreaves test (E), cold allodynia (dry ice) test (F), rotarod test (G). Bars are mean ± S.E.M.; *** indicates significant difference with p<0.001 for groups indicated by the connector line (one-way ANOVA with Tukey post-hoc test).

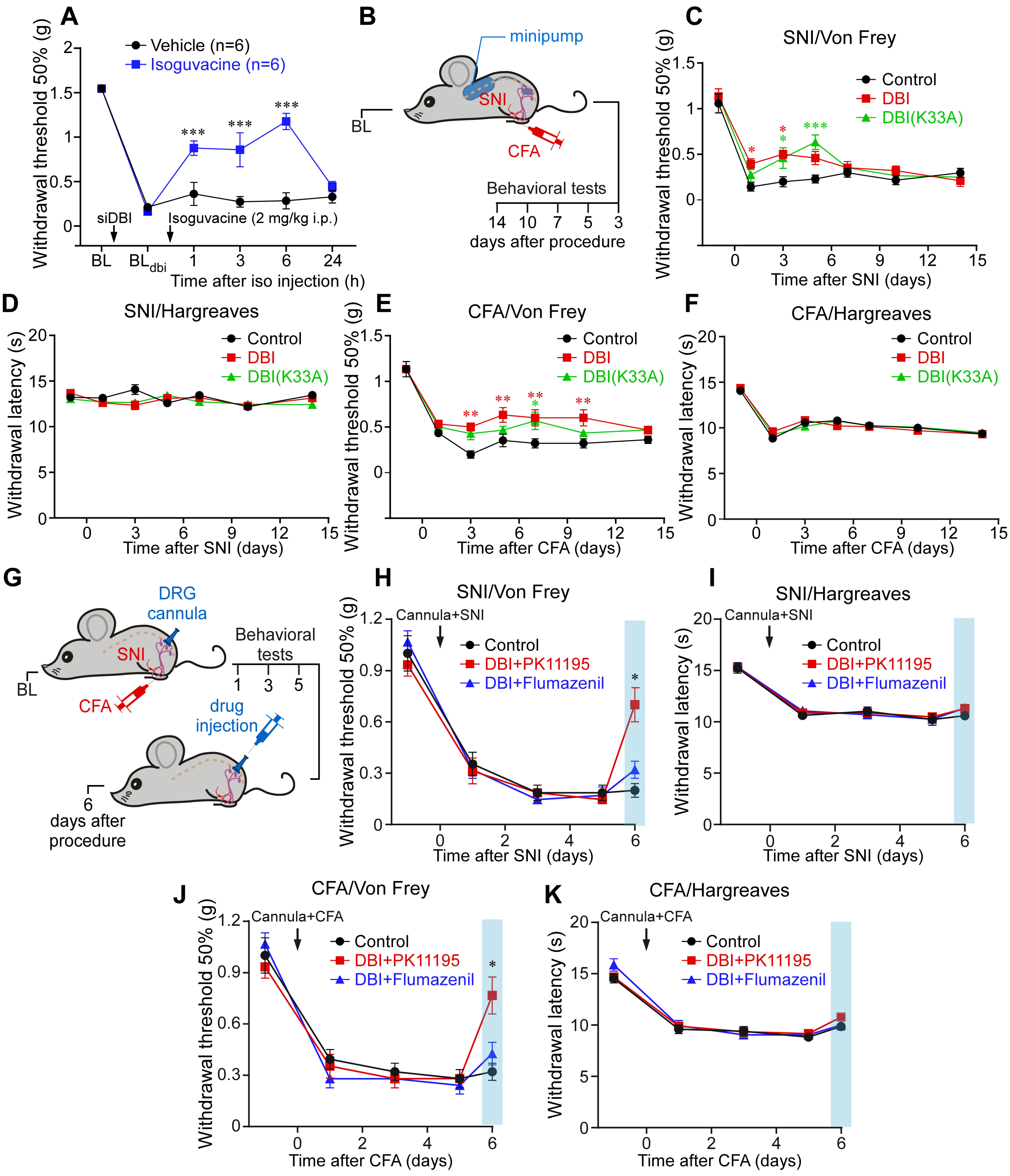

Supplemental Figure 10. The main site of action of DBI in the DRG is the GABA$_A$ receptors. A, Recovery of mechanical hypersensitivity (von Frey test) induced by the intrathecal siRNA knockdown of DBI with the i.p. injection (mouse) of the peripherally-restricted GABA$_A$ agonist, isoguvacine (2 mg/kg; blue), as compared to saline injection (black). The timing of siRNA and isoguvacine injections is indicated by the black arrows. *** indicates significant difference from time-matched saline group (p<0.001; two-way repeated-measures ANOVA with Sidák post-hoc test). **B-F**, Disabling Acetyl-CoA binding site of DBI does not antagonise its anti-nociceptive properties. **B,** Schematic of the experimental timeline for the DRG mini-pump experiments. The DRG minipump implantation was performed and, at the same time, mice received either the hindpaw injection of CFA

(25 μl) or the SNI surgery. Behavioural tests were performed on days 3, 5, 7, 10, and 14 thereafter. **C**, **D**, Mechanical (C) and heat (D) sensitivity was monitored after the SNI induction to the mice implanted with osmotic mini-pumps delivering either DBI (red; 200 μM, 0.5 μl/h) or mutant DBI(K33A) (green; 200 μM, 0.5 μl/h). *, ***, indicate significant difference from time-matched control group (at $p<0.05$, or $p<0.001$, respectively; two-way repeated-measures ANOVA with Tukey's post-hoc test). **E**, **F**, Mechanical (E) and heat (F) sensitivity was monitored after the CFA induction to the mice pre-implanted with osmotic mini-pumps delivering either DBI or mutant DBI(K33A). Other conditions as in panels (B, C) *, **, indicate significant difference from time-matched control group (at $p<0.05$, or $p<0.01$, respectively; two-way repeated-measures ANOVA with Tukey's post-hoc test). **G-K**, benzodiazepine antagonist, flumazenil, but not TSPO antagonist, PK11195, antagonises anti-nociceptive properties of DBI. **G,** Schematic of the experimental. The DRG cannula implantation was performed and, at the same time, mice received either the hindpaw injection of CFA (25 μl) or the SNI surgery. **H**, **I**, Mechanical (B) and heat (C) sensitivity was monitored after the SNI induction to the mice implanted with DRG cannulas. 0.5 hrs before last measurements DBI (200 μM, 2 μl) was co-injected with either PK11195 (red; 200 μM, 2 μl total volume) or flumazenil (blue; 200 μM, 2 μl total volume). *, indicates significant difference from time-matched control group ($p<0.05$; two-way repeated-measures ANOVA with Tukey's post-hoc test). **J**, **K**, Experiments similar to these shown in panels I, J but CFA inflammatory pain model was used instead of SNI. * indicates significant difference from time-matched control group ($p<0.05$; two-way repeated-measures ANOVA with Tukey's post-hoc test).

Movie S1 Light-sheet microscopy of cleared rat lumbar DRG immunolabeled with NF200 (green), peripherin (red) and DBI (white). Staining, iDISCO clearance and imaging was performed as described in (*6*).
