# Supplementary figures and images for: Peripheral gating of mechanosensation by glial diazepam binding inhibitor
Source: J Clin Invest. 2024 Jun 18;134(16):e176227. doi: 10.1172/JCI176227 (PMC11324294; doi:10.1172/JCI176227)

Figure 1B

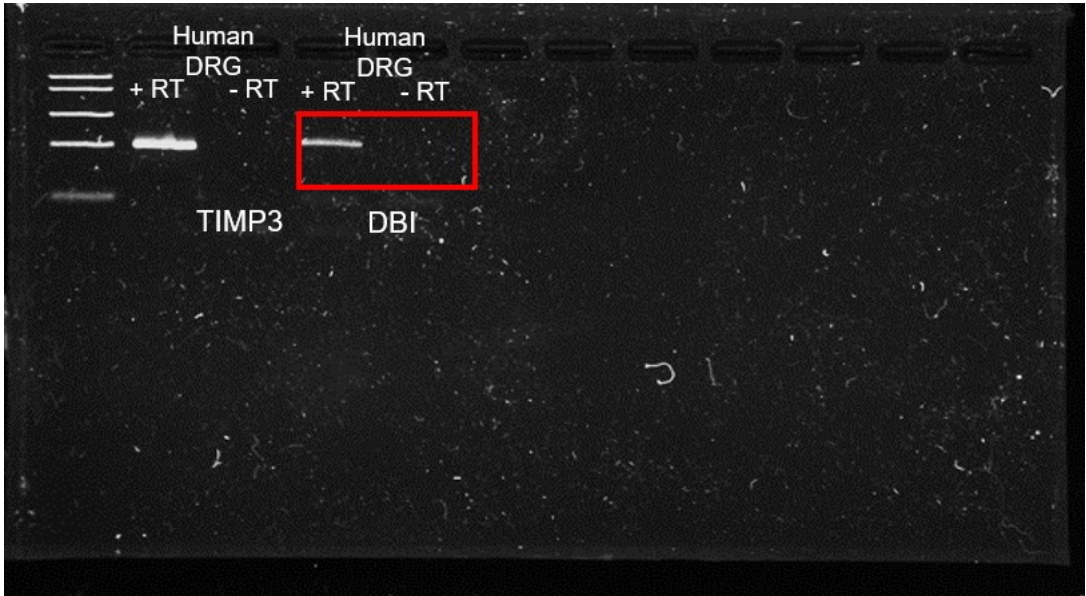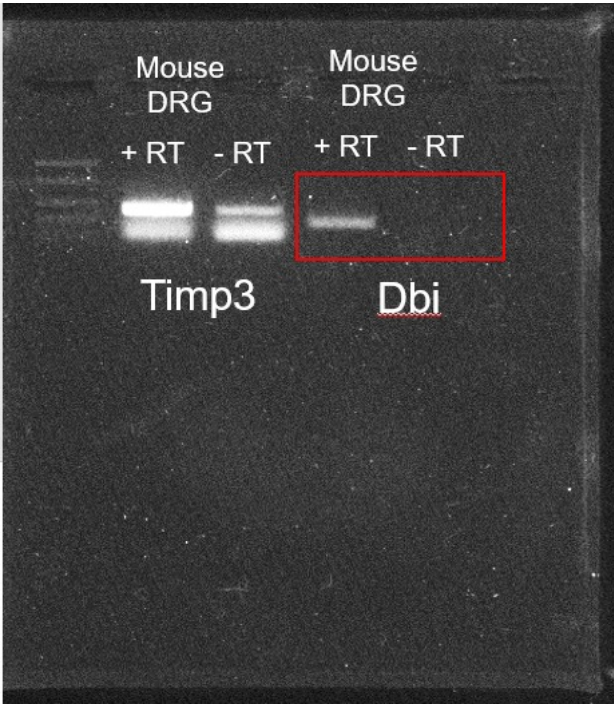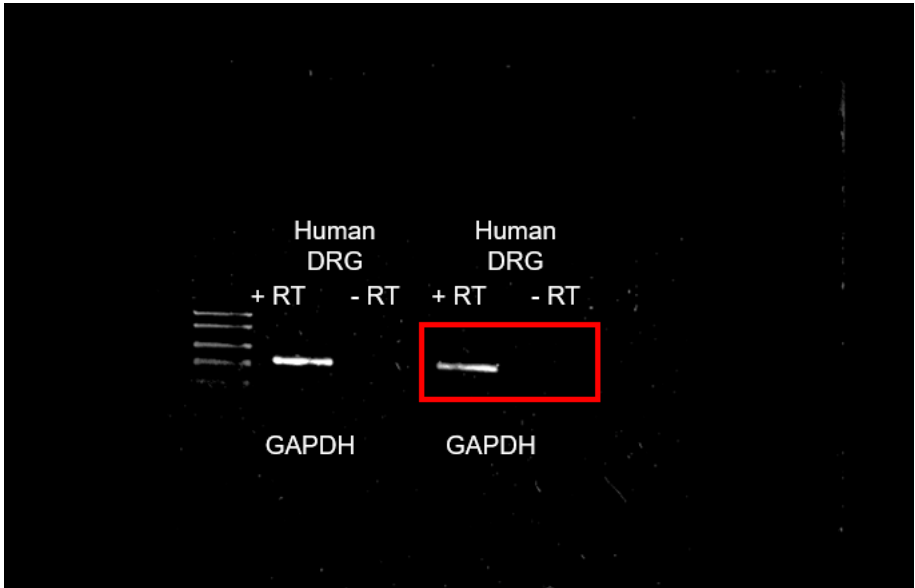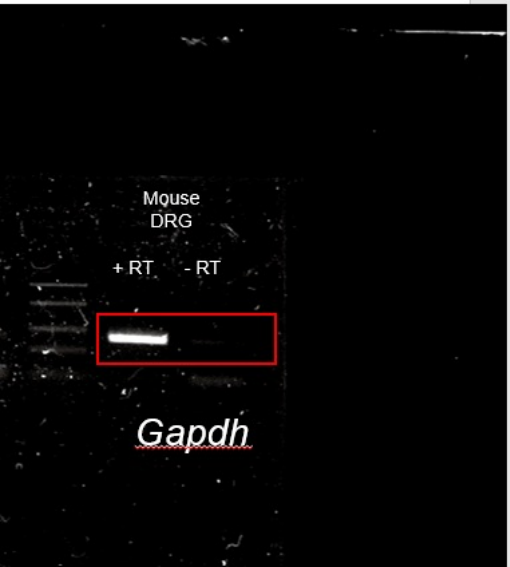

Figure 3B

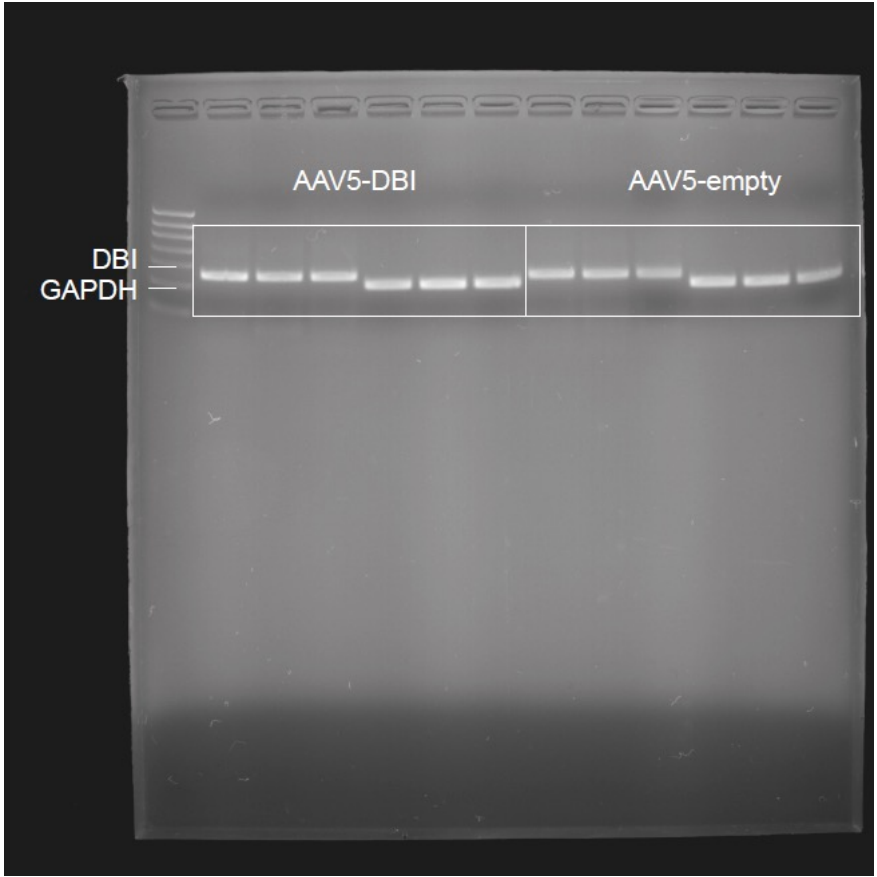

Suppl. Fig. 5C

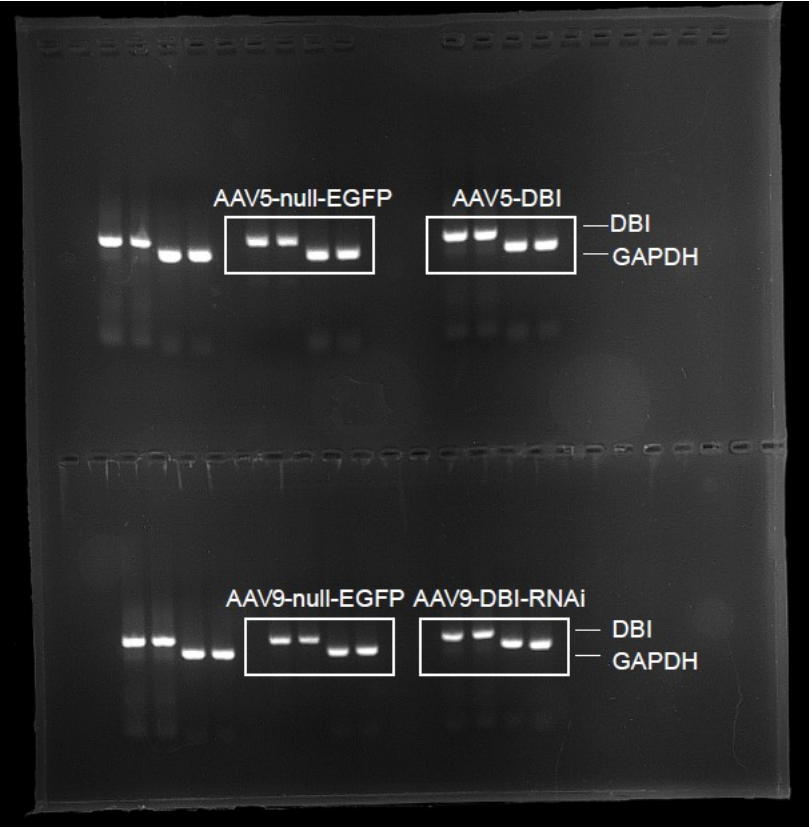

Suppl. Fig. 6B

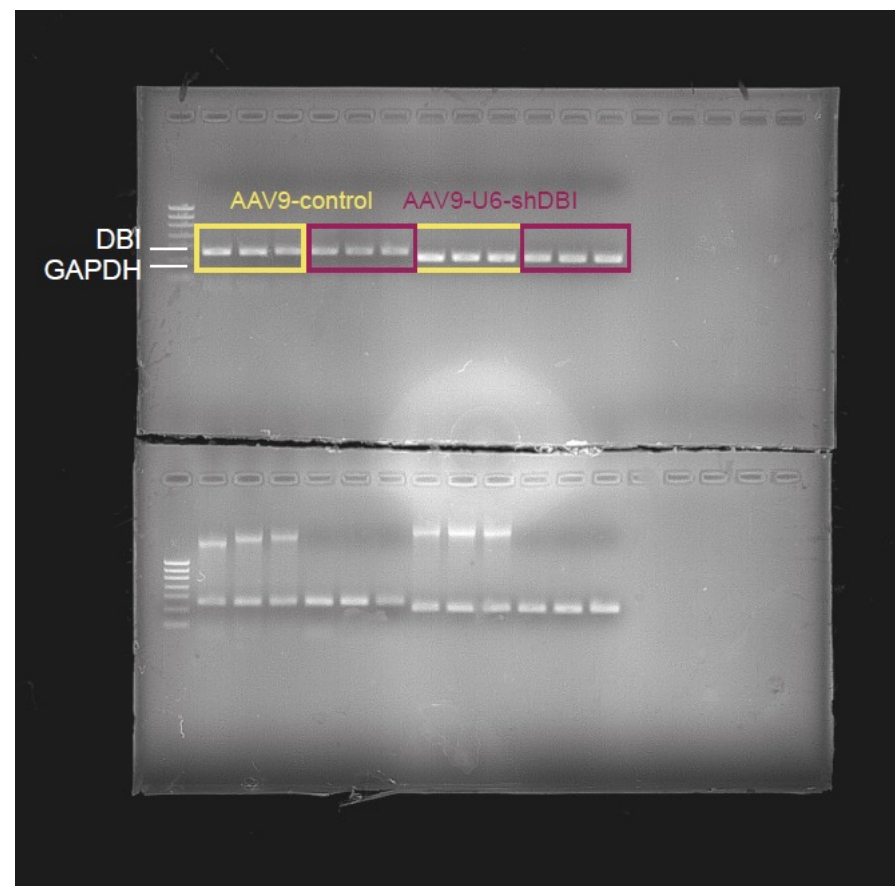

Supplement: Unedited blot and gel images [file jci-134-176227-s120.pdf]
